# Selective and eco-friendly procedures for the synthesis of benzimidazole derivatives. The role of the Er(OTf)_3_ catalyst in the reaction selectivity

**DOI:** 10.3762/bjoc.12.235

**Published:** 2016-11-16

**Authors:** Natividad Herrera Cano, Jorge G Uranga, Mónica Nardi, Antonio Procopio, Daniel A Wunderlin, Ana N Santiago

**Affiliations:** 1 INFIQC-CONICET and Facultad de Ciencias Químicas, Departamento de Química Orgánica, Universidad Nacional de Córdoba, Ciudad Universitaria, Córdoba, 5000 Argentina; 2 Dipartimento di Chimica, Università della Calabria Cubo 12C, 87036-Arcavacata di Rende (CS), Italia; 3 Dipartimento di Scienze della Salute, Università Magna Graecia, Viale Europa, 88100-Germaneto (CZ), Italia; 4 ICYTAC-CONICET and Facultad de Ciencias Químicas, Departamento de Química Orgánica, Universidad Nacional de Córdoba, Ciudad Universitaria, Córdoba, 5000 Argentina

**Keywords:** catalysis, charge density, condensation, erbium(III) trifluoromethanesulfonate, green procedure, heterocycle

## Abstract

An improved and greener protocol for the synthesis of benzimidazole derivatives, starting from *o*-phenylenediamine, with different aldehydes is reported. Double-condensation products were selectively obtained when Er(OTf)_3_ was used as the catalyst in the presence of electron-rich aldehydes. Conversely, the formation of mono-condensation products was the preferred path in absence of this catalyst. One of the major advantages of these reactions was the formation of a single product, avoiding extensive isolation and purification of products, which is frequently associated with these reactions.

Theoretical calculations helped to understand the different reactivity established for these reactions. Thus, we found that the charge density on the oxygen of the carbonyl group has a significant impact on the reaction pathway. For instance, electron-rich aldehydes better coordinate to the catalyst, which favours the addition of the amine group to the carbonyl group, therefore facilitating the formation of double-condensation products.

Reactions with aliphatic or aromatic aldehydes were possible, without using organic solvents and in a one-pot procedure with short reaction time (2–5 min), affording single products in excellent yields (75–99%). This convenient and eco-friendly methodology offers numerous benefits with respect to other protocols reported for similar compounds.

## Introduction

The formation of heterocyclic compounds is a very important task in organic synthesis, mainly because they are present in numerous biologically active compounds and in several natural products [1]. Among them the presence of benzimidazole [2–7] or benzothiazole [8–9] rings in numerous compounds is an important structural element for their biological and medical applications. For example benzimidazoles are widely spread in antiulcer, antihypertensive, antiviral, antifungal, anticancer, and antihistaminic medicines, among others [10–12].

**Scheme 1:**
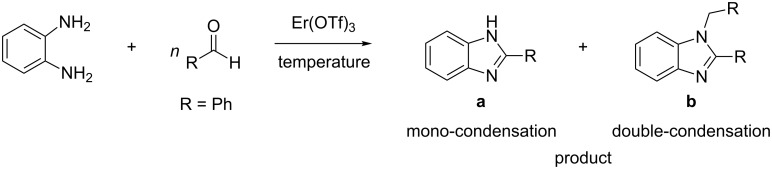
Formation of the benzimidazole core.

One frequently used protocol for the synthesis of benzimidazole derivatives is the coupling of *o*-phenylenediamines with carboxylic acids [13–14]. Another widely used procedure for the same synthesis represents the condensation of *o*-phenylenediamine with aldehydes. The latter approach has become more widely accepted, because of the easy access to a variety of substituted aldehydes. For instance, the reaction between *o*-phenylenediamine and benzaldehyde readily affords benzimidazole derivatives (Scheme 1). However, the reaction is not selective, affording both 2-substituted (**a**) and 1,2-disubstituted benzimidazoles (**b**).

Therefore, the main drawbacks of current protocols for the synthesis of benzimidazoles include the use of expensive reagents, difficulties in the preparation of the catalyst, long reaction times, a narrow scope of substrates, tedious work-up procedures, the use of hazardous organic solvents and lack of selectivity [15–21].

Rare earth metals are economical and readily available from commercial sources and represent useful catalysts in organic synthesis [22]. In particular, erbium(III) promotes environmentally friendly reactions [23–25], and has been successfully applied to the synthesis of natural products [26–28]. For instance, an efficient method for the synthesis of a wide range of 3,3-dimethyl-11-alkyl, or aryl 2,3,4,5-tetrahydro-1*H*-dibenzo[*b*,*e*][1,4]diazepin-1-ones was reported using erbium(III) trifluoromethanesulfonate, Er(OTf)_3_ as catalyst. The reaction comprises a one-pot condensation between *o*-phenylenediamine and 5,5-dimethylcyclohexane-1,3-dione, followed by a Er(OTf)_3_-catalyzed cyclization with diverse alkyl- or arylcarbonyl chlorides [29–30].

In view of these previous applications, our main goal was the development of an environmentally friendly synthetic method, to obtain different derivatives containing the benzimidazole core by a one-pot reaction. Additionally, Er(OTf)_3_ was selected as the catalyst to achieve the selective formation of products in order to avoid tedious work-up and product separation procedures. Moreover, differences in reactivity were investigated by by means of theoretical calculations.

## Results and Discussion

The benzimidazole core was obtained by air oxidative cyclocondensation of *o*-phenylenediamine with benzaldehyde under different conditions. In water and in the presence of Er(OTf)_3_, the diamine and benzaldehyde (1:2 ratio) selectively afforded 1-benzyl-2-phenyl-1*H*-benzimidazole (**1b**) (72% yield), using both microwave irradiation and conventional heating for 15 minutes (Table 1, entries 1 and 3). In the absence of the catalyst, the same reaction afforded a mixture of products **1a** and **1b** using both conditions. Namely, under microwave irradiation, 41% of **1a** and 51% of **1b** were formed (Table 1, entry 2). While, using conventional heating, 52% of **1a** and 40% of **1b** were formed (Table 1, entry 4).

**Table 1:** Comparison of the efficiency of various catalysts, solvents and temperatures in the reaction of *o*-phenylenediamine with benzaldehyde.^a^

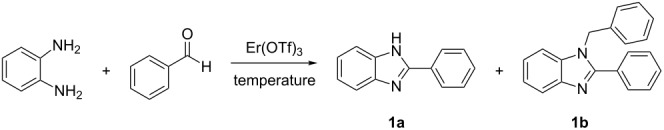						

Entry	Catalyst	Solvent	Temperature (°C)	Time (min)	Yield (%)	References

1	Er(OTf)_3_	H_2_O	MW/120^b^	15	72 (**1b**)^c^	this work
2	–	H_2_O	MW/120^b^	15	51 (**1b**) 41 (**1a**)	this work
3	Er(OTf)_3_	H_2_O	120^b^	15	72 (**1b**)^c^	this work
4^d^	–	H_2_O	120^b^	15	40 (**1b**) 52 (**1a**)	this work
5	Er(OTf)_3_	H_2_O	rt	5	62 (**1b**)^c^	this work
6	Er(OTf)_3_	H_2_O	120	5	74 (**1b**)^c^	this work
7	–	H_2_O	120	5	43 (**1b**) 55 (**1a**)	this work
8	Er(OTf)_3_	ethanol	120	2	91 (**1b**)	this work
9	–	ethanol	120	2	54 (**1b**) 41 (**1a**)	this work
10	Er(OTf)_3_	–	80	2	91 (**1b**)	this work
11^e^	Er(OTf)_3_	–	80	2	90 (**1b**)	this work
12	ErCl_3_·6H_2_O	–	80	15	71 (**1b**) 5 (**1a**)	this work
13	ErCl_3_	–	80	15	89 (**1b**)	this work
14	Yb(OTf)_3_	–	80	60	70 (**1b**)	this work
15	Ce(OTf)_3_	–	80	60	88 (**1b**)	this work
16	SDS	H_2_O	rt	22	98 (**1b**)	[31]
17	LaCl_3_	–	rt	60	99 (**1b**)	[32]
18	SiO_2_/ZnCl_2_	–	rt	20	72 (**1b**)	[33]
19	PHP	H_2_O	50	120	76 (**1b**)	[15]
20	HClO_4_–SiO_2_	ethanol	rt	60	90 (**1b**)	[16]
21	PSSA	H_2_O	rt	35	90 (**1b**)	[34]
22	HSO_3_Cl	2-propanol	rt	108	93 (**1b**)	[35]
23	TMSCl	H_2_O	rt	300	87 (**1b**)	[36]
24	Amberlite IR-120	ethanol/H_2_O	25^f^	132	82 (**1b**)	[37]
25^g^	Er(OTf)_3_	H_2_O	1	5	35 (**1a**) 50 (**1b**)	this work
26^h,d^	–	H_2_O	1	5	92 (**1a**) 8 (**1b**)	this work
27	air	ethanol	rt	540	70 (**1a**)	[38]
28	air	H_2_O	100 °C	240	58 (**1a**)	[39]
29	IBD	dioxane	rt	5	98 (**1a**)	[40]
30	Ru(bpy)3Cl2	methanol	rt	120	95 (**1a**)	[42]
31	Ir(dfppy)_2_(phen)PF_6_	methanol	rt	120	66 (**1a**)	[42]

^a^General reaction conditions: 2 mmol of benzaldehyde and 1 mmol of *o*-phenylenediamine, 10 mol % of Er(OTf)_3_ under conventional heating. ^b^The reaction mixture was heated in a bath at 120 °C using a closed vessel. ^c^Only remaining reactants were observed. ^d^At 40 min the yield of **1b** was 54%. ^e^Under N_2_ atmosphere. ^f^Under sonication. ^g^The amine/aldehyde molar ratio was 1:1.1. ^h^The amine/aldehyde molar ratio was 4:1.

To shorten the reaction time, the catalyzed reaction was carried out during 5 minutes at room temperature. Using these last conditions, the reaction afforded selectively **1b** in 62% yield (Table 1, entry 5). On the other hand, when the reaction was carried out at 120 °C during 5 minutes, with and without catalyst, product **1b** was also formed, with yields of 74% and 43% yield, respectively (Table 1, entries 6 and 7).

Next, different solvents were evaluated aiming at increasing the product yield. When ethanol/water was used as solvent, **1b** was formed together with a small amount of product **1a**. However, changing to ethanol as the solvent, the reaction of diamine with benzaldehyde at 120 °C selectively afforded 91% of **1b** (Table 1, entry 8). Conversely, the reaction without catalyst in ethanol afforded a mixture of products **1a** (41%) and **1b** (54%) (Table 1, entry 9). The highest selectivity towards the double-condensation product **1b** was obtained in the reaction without any solvent at 80 °C. Under these conditions, product **1b** could be isolated in 91% yield after 2 min reaction time (Table 1, entry 10). The use of Er(OTf)_3_ under a N_2_ atmosphere did not change the yield nor the reaction times (Table 1, entry 11). Changing the catalyst to ErCl_3_·6H_2_O, the reaction afforded 71% **1b** with a small amount (5%) of **1a**, after 15 min (Table 1, entry 12). The reaction was more selective using ErCl_3_ during 15 minutes (Table 1, entry 13). The reaction was also carried out with other lanthanides such as Yb(OTf)_3_ and Ce(OTf)_3_, both requiring longer times (60 min) to achieve comparable product yields (Table 1, entries 14 and 15).

Table 1 summarizes these results, comparing our current results with other catalysts previously used in the synthesis of benzimidazole derivatives. For instance, the reaction of *o*-phenylenediamine with aromatic aldehydes using sodium dodecyl sulfate (SDS) as the catalyst gave **1b** in 98% yield. However, the yields were low using aliphatic aldehydes together with SDS as catalyst (Table 1, entry 16) [31]. Conversely, good to moderate yields were observed in reactions between benzaldehyde and *o*-phenylenediamine catalyzed by lanthanum (LaCl_3_) [32]_,_ SiO_2_/ZnCl_2_ [33], polymeric resin-bound hexafluorophosphate ion (PHP) [15], perchloric acid adsorbed on silica gel (HClO_4_–SiO_2_) [16], polystyrene sulfonic acid [34], HSO_3_Cl in 2-propanol [35], trimethylsilyl chloride (TMSCl) [36], or Amberlite (IR-120) [37]. It is worth mentioning that these previously reported catalysts required longer reaction times than those used in our current protocol (Table 1, entries 17–24). Moreover, although other methods are quite satisfactory with regards to reaction yield, many of them were carried out at high temperatures, or require expensive catalysts. Furthermore, several previously reported reactions employed organic solvents, which are not environmentally friendly. Thereby, we propose the use of Er(OTf)_3_ as catalyst to provide an eco-friendly, economical and easy to work-up procedure for the synthesis of 1,2-disubstituted benzimidazoles, which can be afforded in only two minutes.

In order to selectively obtain 2-phenyl-1*H*-benzimidazole (**1a**), the reaction was carried out using *o*-phenylenediamine and benzaldehyde (1:1.1 ratio) in water, at 1 °C, adding 10 mol % Er(OTf)_3_. Under these conditions, 35% of 2-phenyl-1*H*-benzimidazole (**1a**) and 50% of 1-benzyl-2-phenyl-1*H*-benzimidazole (**1b**) were obtained after 5 min reaction (Table 1, entry 25). When this reaction was performed without catalyst, 92% of **1a** and 8% of **1b** were observed using a 4:1 amine/aldehyde ratio (Table 1, entry 26). This ratio favored the fast cyclization, affording excellent yields of mono-condensation product **1a**.

Several reactions between benzaldehyde and *o*-phenylenediamine to obtain 2-phenyl-1*H*-benzimidazole (**1a**) are known. However, they afforded moderate yields requiring longer reaction times in the presence of air (Table 1, entries 27 and 28) [38–39]. Product **1a** was also obtained in a shorter reaction time using hypervalent iodine as oxidant and dioxane as solvent [40–41] or in the presence of [Ru(bpy)_3_Cl_2_] or Ir(dfppy)_2_(phen)PF_6_ as catalysts [42] (Table 1, entries 29–31). The major disadvantage of these methods, however, is the cost of these catalysts.

Thus, comparing previous reports with our current method, it is concluded that the use of Er(OTf)_3_ as catalyst provides many advantages over previous ones such as it makes use of an economical, eco-friendly and recyclable catalyst, excellent yields in short reaction times, a simple procedure, short reaction times, and an easy work-up.

**Scheme 2:**
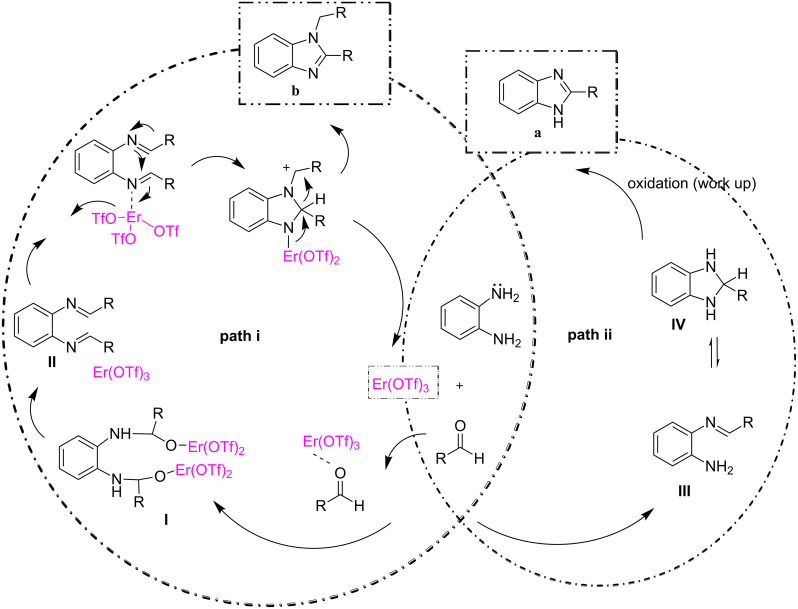
Proposed mechanism for the formation of 1,2-disubstituted benzimidazoles **b** and 2-substituted benzimidazoles **a**.

In addition to the above mentioned advantages we observed that erbium is not involved in the formation of **1a**, but it catalyzes the formation of **1b**. The mechanism for the formation of **1b** using lanthanum catalysts (LaCl_3_) was reported by Zhang et al. [32]. Considering all the evidences, and the essential role of Er(OTf)_3_ on the selectivity between **1a** and **1b** observed in this work, two reaction pathways are proposed and shown in Scheme 2: (i) through bisimine rearrangement (path i) and (ii) through a monoamine cyclocondensation–aminal/immonium rearrangement (path ii).

In path i, when the aldehyde approaches Er(OTf)_3_, the carbonyl carbon of the aldehyde becomes highly reactive toward the nucleophilic attack of *o*-phenylenediamine, generating dibenzylidenediamine **I**. Consequently, the 1,2-disubstituted benzimidazole (**b**) will be formed through bisimine **II**, under catalytic action of the Lewis acid Er(OTf)_3_. Thus, the catalyst acts as an effective electrophilic activating agent for the formation of the bisimine and promotes the subsequent steps (intramolecular nucleophilic attack and the following 1,3-hydride shift), finally affording the 1,2-disubstituted imidazoles (**b**).

In contrast, path ii is a non-catalyzed reaction. In path ii, when the diamine reacts with the aldehyde, a monoimine **III** is formed. The latter intermediate undergoes an intramolecular nucleophilic attack on the C=N, leading to the formation of the imidazoline intermediate **IV**. This intermediate finally affords 2-substituted benzimidazole **1a**. Thus, the presence of erbium determines the reaction pathway (either i or ii), controlling the selective formation of 1,2-disubstituted vs 2-substituted benzimidazole. It is worth to remark that the presence of the carbonyl hydrogen in the aldehyde is necessary for the formation of the benzimidazole core. On the contrary, the reaction of the diamine with ketones affords benzodiazepine as products [29–30].

Next, we investigated the general applicability of our method in the reaction of *o*-phenylenediamine with several substituted aldehydes using the optimized conditions towards products **1a** or **1b**, respectively. For this, the best conditions to selectively obtain the double-condensation product **1b** (Table 1, entry 10) were chosen and a family of 1,2-disubstituted benzimidazoles was successfully synthesized. The results are listed in Table 2.

**Table 2:** Synthesis of 1,2-disubstituted benzimidazoles.^a^

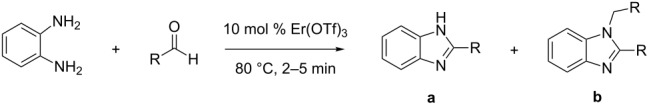				

Entry	R	Time (min)	Product	Yield (%)

1^b^	Ph	2	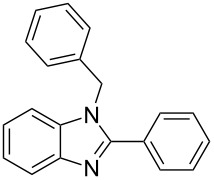 **1b**	91
2^c^	4-H_3_COC_6_H_4_	2	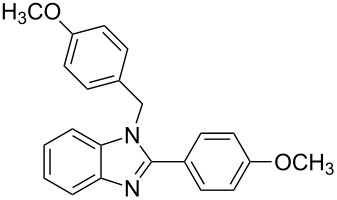 **2b**	85
3^d^	4-CH_3_C_6_H_4_	2	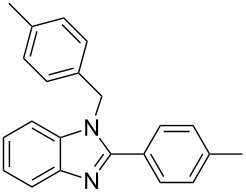 **3b**	83
4^e^	CH_3_CH_2_	2	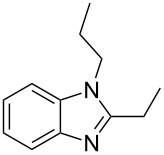 **4b**	96
5^f^	H_3_C	2	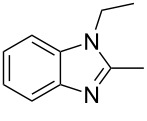 **5b**	98
6	C_6_H_5_-CH_2_	2	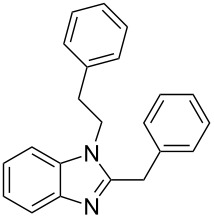 **6b**	97
7^g^	4-ClC_6_H_4_	2–5	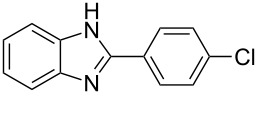 **7a**	78
8^g^	4-NO_2_C_6_H_4_	2–5	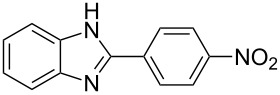 **8a**	79
9^g^	4-CNC_6_H_4_	2–5	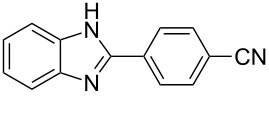 **9a**	82

^a^General reaction conditions: 1 mmol of benzaldehyde and 0.5 mmol of *o*-phenylenediamine, 10 mol % of Er(OTf)_3_ under conventional heating at 80 °C for the indicated time. ^b^With 9% of **1a**. ^c^With 15% of **2a**. ^d^With 17% of **3a**. ^e^With 4% of **4a**. ^f^With 2% of **5a**. ^g^Product **b** was not detected. Similar yields were obtained without catalyst.

The reactions of *o*-phenylenediamine with electron-rich aldehydes, such as 4-CH_3_OC_6_H_4_CHO, 4-CH_3_C_6_H_4_CHO, CH_3_CH_2_CHO, CH_3_CHO and 4-C_6_H_5_-CH_2_CHO (Table 2, entries 2–6) afforded the corresponding 1,2-disubstituted benzimidazoles **2b–7b** in good yields (over 83%) under the optimized conditions. However when aldehydes containing electron-withdrawing groups, such as 4-ClC_6_H_4_CHO, 4-NO_2_C_6_H_4_CHO and 4-CNC_6_H_4_CHO were used, unexpected products were observed (Table 2, entries 7–9). Instead of double-condensation products **b**, the corresponding mono-condensation products **7a**–**9a** were formed in excellent yields. The same products were obtained in comparable yields without the use of catalyst.

These results clearly show that the electronic effects of the substituents present in the aldehydes play a significant role in the reaction pathway. The 1,2-disubstituted benzimidazoles were obtained when electron-rich aldehydes were used, while 2-monosubstituted benzimidazoles were obtained from the reaction with electron-deficient aldehydes under the same conditions.

To shed light on this observation, we decided to carry out theoretical calculations, using the BPW91 functional at 6-31+G* level, as implemented in Gaussian 09 [43]. In order to evaluate the effect of the substituent on the reactivity of aldehydes, we used a molecular descriptor based on the electronic properties of the carbonyl group. These properties could determinate the affinity between the aldehyde and the catalyst.

**Figure 1:**
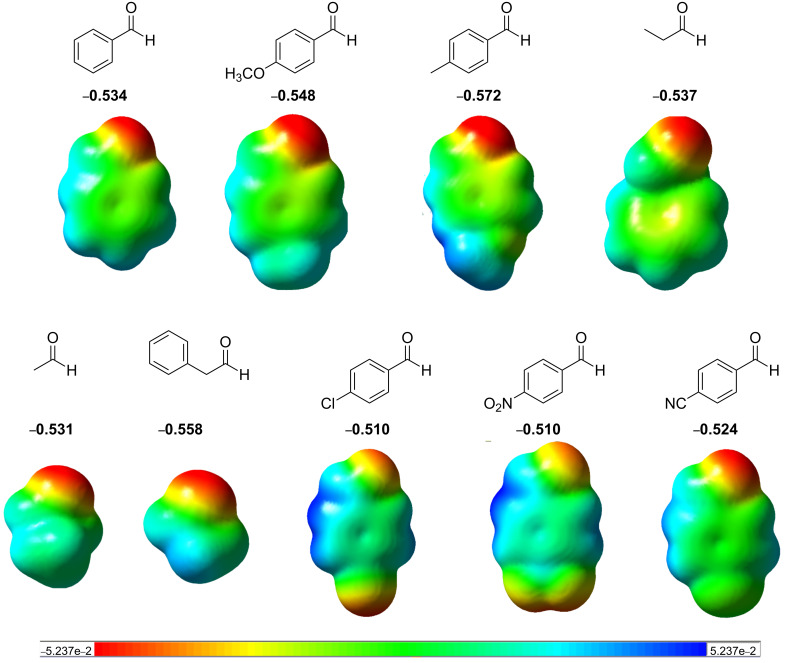
ESP maps and charge density on carbonylic oxygen atoms for the studied aldehydes obtained at the BPW91/6-31+G* level. All maps used consistent surface potential ranges (−0.05237 (red) to 0.05237 (blue)) and an isovalue of electron density of 0.0004. All values are expressed in atomic units.

Geometries were optimized for all aldehydes and electrostatic potential (ESP) population analyses were done to obtain the charge density on the carbonyl group. The calculated charge density on the oxygen of carbonyl group could indicate the reactivity of these aldehydes. The greater the charge density on the oxygen, the greater the affinity of it to erbium, enabling the formation of 1,2-disubstituted benzimidazoles (Scheme 2, path i). As it can be seen from Figure 1, the charge density at the oxygen of the carbonyl group is a well-suited molecular descriptor for the behavior of the aldehyde.

Our current results show that the charge density for aldehydes containing electron-donating or aliphatic groups varies from −0.57 to −0.53, while the corresponding densities for aldehydes containing electron-withdrawing groups were found to be in a range from −0.52 to −0.51. The aldehydes containing electron-donating or aliphatic groups show a higher density of negative charge on the oxygen atom than aldehydes containing electron-withdrawing groups (Figure 1). As a consequence, electron-rich aldehydes coordinate better with the catalyst, promoting the addition of the amine group to the carbonyl group, and affording double-substitution products (Scheme 2, path i). Conversely, aldehydes substituted with electron-withdrawing groups do not coordinate well due to their lower density of negative charge on the oxygen atom (Figure 1). In the latter case, the formation of mono-condensation products is favored without the intervention of the catalyst (Scheme 2, path ii). These results are consistent with our experimental results.

Next the selectivity towards the mono-condensation products **a** was investigated (Table 3) using the best conditions identified for the synthesis of 2-phenylbenzimidazole (**1a**, Table 1, entry 21). As it can be seen from Table 3, good reaction yields (>80%) were obtained with aldehydes containing both, electron-donating groups (Table 3, entries 2–6) and electron-withdrawing groups (Table 3, entries 7-8) at low temperature using short reactions times. Thus, the new procedure is highly versatile for the selective synthesis of 2-substituted benzimidazoles of general type **a**.

**Table 3:** Synthesis of 2-substituted benzimidazoles **a**.^a^

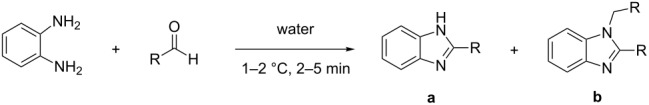				

Entry	R	Time (min)	Product	Yield (%)

1^b^	Ph	2	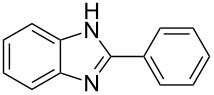 **1a**	92
2^c^	4-H_3_COC_6_H_4_	2	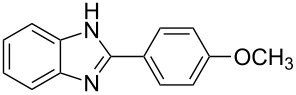 **2a**	99
3^d^	4-CH_3_C_6_H_4_	2	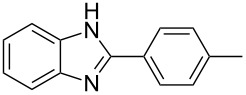 **3a**	94
4^e^	CH_3_CH_2_	1	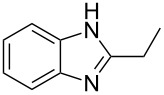 **4a**	96
5^f^	H_3_C	1	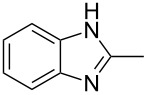 **5a**	97
6^g^	C_6_H_5_-CH_2_	2	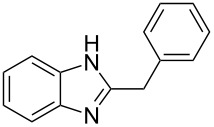 **6a**	91
7^h^	4-ClC_6_H_4_	5	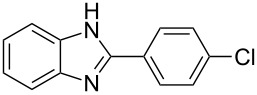 **7a**	81
8^h^	4-NO_2_C_6_H_4_	5	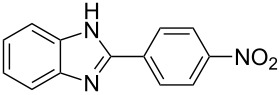 **8a**	85

^a^General reaction conditions: 0.5 mmol of benzaldehyde and 2 mmol of *o*-phenylenediamine at 1–2 °C in 2–5 minutes without catalyst. ^b^With 8% of **1b**. ^c^Product **b** was not detected. ^d^With 5% of **3b**. ^e^With 4% of **4b**. ^f^With 3% of **5b** and **7b**, respectively. ^g^With 9% of **6b**. ^h^Product **b** was not detected.

## Conclusion

We reported a practical and environmentally friendly one-pot method for the simple and selective synthesis of 1,2-disubstituted or 2-substituted benzimidazoles, starting from *o*-phenylenediamine in the presence of aromatic or aliphatic aldehydes. The use of Er(OTf)_3_ as commercially available and easily recyclable catalyst promoted the synthesis of 1,2-disubstituted benzimidazoles. Other lanthanides also catalyzed this reaction but required longer reaction times. On the other hand, 2-substituted benzimidazoles were selectively obtained in high yield and short reaction times by the reaction of phenylenediamine with various aldehydes at low temperature (1–2 °C) or at 80 °C in case of electron-deficient aldehydes.

The observed different reactivity, leading to the formation of either mono- or double-condensation products, was explained on the basis of calculated charge densities located on the carbonyl group that is necessary for the coordination of the catalyst. We suggest that the calculated charge densities on the oxygen could indicate the reactivity of these aldehydes. Moreover, the theoretical results predict that a charge density on the oxygen higher than −0.52 favors the coordination to the catalyst, therefore affording double-condensation products, which is in full agreement with the experimental results of this work.

The proposed methodology possesses numerous advantages over previously reported methods, such as high product yields (83–98%), environmentally friendly and mild reaction conditions, short reaction times (2–5 min), high selectivity and broad application. This method could help to produce bioactive compounds using an environmentally friendly procedure.

## Supporting Information

File 1 Experimental section, spectroscopical data and XYZ coordinates for all compounds.
