# Supplementary material for: Selective and eco-friendly procedures for the synthesis of benzimidazole derivatives. The role of the Er(OTf)3 catalyst in the reaction selectivity
Source: Beilstein J Org Chem. 2016 Nov 16;12:2410–9. doi: 10.3762/bjoc.12.235 (PMC5238590; doi:10.3762/bjoc.12.235)
Supplement: File 1 — Experimental section, spectroscopical data and XYZ coordinates for all compounds. [file Beilstein_J_Org_Chem-12-2410-s001.pdf]

Supporting Information

for

Selective and eco-friendly procedures for the synthesis of benzimidazole derivatives. The role of the $Er(OTf)_3$ catalyst in the reaction selectivity

Natividad Herrera Cano[1], Jorge G. Uranga[1], Mónica Nardi[2], Antonio Procopio[3], Daniel A. Wunderlin[4] and Ana N. Santiago*[1$]

Address: [1]INFIQC-CONICET and Facultad de Ciencias Químicas, Departamento de Química Orgánica, Universidad Nacional de Córdoba, Ciudad Universitaria, Córdoba, 5000 Argentina, [2]Dipartimento di Chimica, Università della Calabria Cubo 12C, 87036-Arcavacata di Rende (CS), Italia, [3]Dipartimento di Scienze della Salute, Università Magna Graecia, Viale Europa, 88100-Germaneto (CZ), Italia and [4]ICYTAC-CONICET and Facultad de Ciencias Químicas, Departamento de Química Orgánica, Universidad Nacional de Córdoba, Ciudad Universitaria, Córdoba, 5000 Argentina

Email: Ana N. Santiago - santiago@fcq.unc.edu.ar

[*]Corresponding Author

[$]Tel: +54 351 5353867, extension 53314

Experimental section, spectroscopical data and XYZ coordinates for all compounds

Table of Content	Page N^o
I. Experimental Section	S2
II. Spectra	
^{1}H & ^{13}C NMR spectra of 2-phenyl-1*H*-benzimidazole (**1a**)	S8
^{1}H & ^{13}C NMR spectra of 1-benzyl-2-phenyl-1*H*-benzimidazole (**1b**)	S9
^{1}H & ^{13}C NMR spectra of 2-(4-methoxyphenyl)-1*H*-benzimidazole (**2a**)	S10
^{1}H & ^{13}C NMR spectra of 1-(4-methoxybenzyl)-2-(4-methoxyphenyl)-1*H*-benzimidazole (**2b**)	S11
^{1}H & ^{13}C NMR spectra of 2-(4-methylphenyl)benzimidazole (**3a**)	S12
^{1}H & ^{13}C NMR spectra of 1-(4-methylbenzyl)-2-(4-methylphenyl)-1*H*-benzimidazole (**3b**)	S13
^{1}H & ^{13}C NMR spectra of 2-ethyl-benzimidazole (**4a**)	S14
^{1}H & ^{13}C NMR spectra of 2-ethyl-1-propyl-1*H*-benzimidazole (**4b**)	S15
^{1}H & ^{13}C NMR spectra of 2-methyl-1*H*-benzimidazole (**5a**)	S16
^{1}H & ^{13}C NMR spectra of 1-ethyl-2-methyl-1*H*-benzimidazole (**5b**)	S17
^{1}H & ^{13}C NMR spectra of 2-benzyl-1*H*-benzo[d]imidazole (**6a**)	S18
^{1}H & ^{13}C NMR spectra of 2-benzyl-1-phenethyl-1*H*-benzo[d]imidazole (**6b**)	S19
^{1}H & ^{13}C NMR spectra of 2-(4-chlorophenyl)benzimidazole (**7a**)	S20
^{1}H & ^{13}C NMR spectra of 2-(4-nitrophenyl)benzimidazole (**8a**)	S21
^{1}H & ^{13}C NMR spectra of 4-(1*H*-1,3-benzimidazol-2-yl)benzonitrile (**9a**)	S22
III. Cartesian coordinates for aldehydes	S23
IV. References	S27

I. Experimental Section

Chemicals and Materials.

All chemicals and solvents were purchased from common commercial sources and used as received without any further purification. ^{1}H and ^{13}C-NMR spectra were recorded on a High Resolution Bruker Advance 400 NMR Spectrometer (working frequency 400 MHz), at room temperature in $CDCl_3$ or DMSO-d_6 (Aldrich). High resolution mass spectra were recorded on a Bruker, Micro QTOF II equipment, operated with an ESI source in (positive/negative) mode, using nitrogen as nebulizing and drying gas and sodium formiate (10 mM) as internal calibration.

General procedures

General experimental procedure for the synthesis of 1,2-disubstituted benzimidazoles. *o*-Phenylenediamine (0.5 mmol, 0.054 g) and $Er(OTf)_3$ (0.05 mmol, 0.031 g) were added to the aldehyde (1 mmol). Solid aldehydes were dissolved in 2 mL of ethanol. Liquid aldehydes were used without solvent. The reaction mixture was stirred at 80 °C for 2 minutes. The crude product was extracted with dichloromethane and water. The organic extract was analyzed by GC–MS, and the products were isolated by radial chromatography, eluting with hexane/ethyl acetate (70:30). Solid products were recrystallized from ethanol. The use of green solvents such as cyclopentyl methyl ether or methyl *tert*-butyl ether in the work-up is possible, but slightly decreased the product yields from 91% to 88 and 85%, respectively.

General experimental procedure for the synthesis of 2-substituted benzimidazoles. *o*-Phenylenediamines (2 mmol, 0.216 g) and aldehyde (0.5 mmol) were added to the 5 mL of water. The reaction was stirred at 1–2 °C for 2–5 minutes. The crude reaction mixture was extracted with dichloromethane and water. The organic extract was analysed by GC–MS. Products were isolated by radial chromatograph,y eluting with hexane/ethyl acetate (85:15).

2-Phenyl-1*H*-benzimidazole (1a): Pale yellow solid; m.p. 293-295°C.

^{1}H NMR (400 MHz, DMSO-d$_6$) δ ppm (J, Hz): 12.92 (br s, 1H), 8.18 (d, J = 9.0 Hz, 2H), 7.67 (d, J = 7.2 Hz, 1H), 7.56-7.49 (m, 4H), 7.22-7.19 (m,2H); ^{13}C NMR (400 MHz, DMSO-d$_6$) δ ppm: 151.3, 143.8, 135.0, 130.2, 129.9, 129.0, 126.5, 122.6, 121.8, 118.2, 111.4. HRMS m/z (ESI) [M+H] calcd. for $C_{13}H_{11}N_2$ 195.0917 found: 195.0916 [1].

1-Benzyl-2-phenyl-1*H*-benzimidazole (1b): White solid; m.p. 132–134 °C. ^{1}H NMR (400 MHz, $CDCl_3$, δ ppm (J, Hz): 7.87 (d, J=7.8 Hz, 1 H), 7.68 (d, J=7.7 Hz, 2 H), 7.47–7.45 (m, 3 H), 7.33–7.22 (m, 6 H), 7.11 (d, J=6.7 Hz, 2 H), 5.46 (s, 2 H); ^{13}C NMR (400 MHz, $CDCl_3$) δ ppm: 154.1, 143.1, 136.3, 136.0, 130.0, 129.8, 129.2, 129.0, 128.7, 127.7, 125.9, 122.9, 122.6, 119.9, 110.4, 48.3. Anal. Calcd for $C_{20}H_{16}N_2$: C, 84.50; H, 5.63; N, 9.85 Found: C, 84.51; H, 5.69; N, 9.80 [2].

2-(4-Methoxyphenyl)-1*H*-benzimidazole (2a): Pale yellow solid; m.p. 224–225 °C. ^{1}H NMR (400 MHz, DMSO-d$_6$) δ ppm (J, Hz): 12.76 (br s, 1H), 8.13 (d, J = 7.2 Hz, 2H), 7.56 (s, 2H), 7.17-7.10 (m, 4H), 3.83 (s, 3H); ^{13}C NMR (400 MHz, DMSO-d$_6$) δ ppm: 160.6, 151.4, 128.0, 122.7, 121.7, 114.4, 114.3, 111.2, 55.3, 14.0. HRMS (ESI) Calc. for $C_{14}H_{13}N_2O$ $[M+H]^+$: 225.1022, found: 225.1021 [1].

1-(4-Methoxybenzyl)-2-(4-methoxyphenyl)-1*H*-benzimidazole (2b): Brown solid; m.p. 129-131 °C. ^{1}H NMR (400 MHz, DMSO-d$_6$) δ ppm (J, Hz): 7.83 (d, J = 8.6 Hz, 1H) 7.63 (d, J = 8.6 Hz, 2H), 7.44 (d, J = 8.5 Hz, 1H), 7.25−7.19 (m, 2H), 7.09 (d, J = 8.6 Hz, 2H), 6.94 (d, J = 8.6 Hz, 2H), 6.85 (d, J = 8.6 Hz, 2H), 5.38 (s, 2H), 3.84 (s, 3H), 3.78 (s, 3H); ^{13}C NMR (400 MHz, DMSO-d$_6$) δ ppm: 160.9, 159.1, 154.1,

143.1, 138.7 136.1, 130.7, 128.5, 128.3, 127.2, 122.8, 122.6, 122.4, 119.7, 114.2, 110.4, 55.3, 55.4, 47.9, 39.7. HRMS m/z (ESI) [M+H] calcd for $C_{22}H_{21}N_2O_2$: 345.1603, found: 345.1591 [2].

2-(4-Methylphenyl)-1*H*-benzimidazole (3a): White solid; m.p. 276–278 °C. ^{1}H NMR (400 MHz, DMSO-d_6) δ ppm (J, Hz): 12.85 (br s,1H), 8.07 (d, J = 7.2 Hz, 2H), 7.63 (s, 1H), 7.52 (s, 1H), 7.36 (d, J = 6.8 Hz, 2H), 7.19 (s, 2H), 2.37 (s, 3H); ^{13}C NMR (400 MHz, DMSO-d_6) δ ppm: 151.4, 143.8, 139.6, 135.0, 129.5, 127.5, 126.4, 122.4, 121.6, 118.7, 111.2, 21.0. HRMS-ESI (*m/z*): $[M+Na]^+$ calcd. for $C_{14}H_{12}N_2Na$ 231.0898; found: 231.0895 [3].

1-(4-Methylbenzyl)-2-(4-methylphenyl)-1*H*-benzimidazole (3b): White solid; m.p. 129-130 °C. ^{1}HNMR (400 MHz, $CDCl_3$) δ ppm (J, Hz): 8.03 (d, J = 8 Hz, 1H), 7.74 (d, J =7.7 Hz, 2H), 7.31−7.18 (m, 6H), 7.13 (d, J = 7.7 Hz, 2H), 7.99 (d, J =7.7 Hz, 2H), 5.42 (s, 2H), 2.65 (s, 3H), 2.40 (s, 3H). ^{13}C NMR (400 MHz, $CDCl_3$) δ ppm: 136.2, 135.8, 130.1, 129.3, 129.1, 128.8, 127.8, 125.9, 123.2, 122.9, 119.9, 110.5, 96.1, 77.6, 76.9, 76.3, 47.8, 47.6, 21.7. Anal. Calcd for $C_{22}H_{20}N_2$: C, 84.58; H, 6.45; N, 8.97. Found: C, 84.69; H, 6.48; N, 8.97 [2].

2-Ethyl-1*H*-benzimidazole (4a): Pale yellow solid; m.p. 176-178°C. ^{1}H NMR (400 MHz, $CDCl_3$) δ ppm (J, Hz): 11.50 (s, 1H), 7.57-7.54 (d, 2H, J = 9.20 Hz), 7.22–7.19 (d, 2H, J = 9.20 Hz), 3.05-2.99 (m, 2H), 1.46-1.42 (m, 3H). ^{13}C NMR (400 MHz, $CDCl_3$) δ ppm: 156.8, 138.6, 130.3, 114.6, 22.6, 12.4. HRMS (ESI) Calc. for $C_9H_{11}N_2$ $[M+H]^+$: 147.0922, found: 147.0921 [4].

2-Ethyl-1-propyl-1*H*-benzimidazole (4b): Yellow oil. ^{1}H NMR (400 MHz, $CDCl_3$), δ ppm (J, Hz): 7.39-7.24, (d, 4H, J= 9.18 Hz), 4.18-4.14 (m, 2H), 3.15-3.11 (m, 2H), 1.92-1.89 (m, 2H), 1.58-1.54 (m, 3H), 1.02-0.90 (m, 3H). ^{13}C NMR (400 MHz, $CDCl_3$) δ ppm: 158.0, 141.7, 134.7, 122.1, 121.1, 120.1, 107.6, 47.2, 20.1, 18.5, 10.3, 9.9. HRMS (ESI) Calc. for $C_{12}H_{17}N_2$ $[M+H]^+$: 189.1392, found: 189.1393 [5].

2-Methyl-1*H*-benzimidazole (5a): Pale yellow solid; m.p. 173-175 °C. ^{1}H NMR (400 MHz, $CDCl_3$), δ ppm (J, Hz): 11.59 (s, 1H), 7.56-7.55 (d, 2H, J = 9.20 Hz), 7.22–7.21 (d, 2H, J = 9.20 Hz), 2.67 (s, 3H). ^{13}C NMR (400 MHz, $CDCl_3$) δ ppm: 148.5, 138.1, 122.3, 114.4, 14.3. HRMS (ESI) Calc. for $C_8H_8N_2Na$ $[M+Na]^+$: 155.0585, found: 155.0583 [6].

1-Ethyl-2-methyl-1*H*-benzimidazole (5b): Brown solid; m.p. 47.9–49.8 °C. ^{1}H NMR (400 MHz, $CDCl_3$), δ ppm (J, Hz): 7.71-7.25, (m, 4H), 4.22-4.16 (s, 2H), 2.64 (s, 3H), 1.44-1.41 (s, 3H). ^{13}C NMR (400 MHz, $CDCl_3$) δ ppm: 151.0, 142.6, 134.6, 121.9, 121.7, 119.0, 108.9, 38.5, 14.9, 13.7. HRMS (ESI) Calc. for $C_{10}H_{13}N_2$: $[M+H]^+$: 161.1079, found: 161.1069 [5].

2-Benzyl-1*H*-benzimidazole (6a): Pale yellow solid; m.p. 184–186 °C. ^{1}H NMR (400 MHz, $CDCl_3$), δ ppm (J, Hz): 7.50-7.18 (m, 9H), 4.25 (s, 2H). ^{13}C NMR (400 MHz, $CDCl_3$) δ ppm: 162.7, 153.5, 137.4, 128.5, 128.4, 128.3, 128.2, 128.1, 122.1, 33.6. HRMS (ESI) Calc. for $C_{14}H_{13}N_2$ $[M+H]^+$: 209,1079, found: 209.1073 [4].

2-Benzyl-1-phenethyl-1*H*-benzimidazole (6b): Yellow oil. ^{1}H NMR (400 MHz, $CDCl_3$), δ ppm (J, Hz): 7.78 (s, 2H), 7.28-7.25 (m, 10 H), 7.00 (s, 2H), 4.15 (s, 2 H), 3.94 (s, 2H), 2.80-2.76 (m, 2H). ^{13}C NMR (400 MHz, $CDCl_3$) δ ppm: 153.2, 142.7, 137.8, 136.4, 136.1, 135.1, 128.9, 128.6, 128.4, 127.0, 126.4, 122.4, 122.0, 120.0, 109.4, 65.8, 35.6, 34.4. HRMS (ESI) Calc. for $C_{22}H_{21}N_2$ $[M+H]^+$: 314.1704, found: 314.1742 [4].

2-(4-Chlorophenyl)-1*H*-benzimidazole (7a): Pale yellow solid; m.p. 295-297 °C. ^{1}H NMR (400 MHz, DMSO-d_6) δ ppm (J, Hz): 12.97 (br s, 1H), 8.19 (d, *J* = 8.4 Hz, 2H), 7.68 (d, J = 7.8 Hz, 1H), 7.63 (d, J = 8.4 Hz, 2H), 7.54 (d, J = 7.8 Hz, 1H), 7.23-7.20 (m, 2H); ^{13}C NMR (400 MHz, DMSO-d_6) δ ppm: 150.1, 143.7, 135.0, 134.4, 129.0, 128.7, 128.1, 122.7, 121.8, 118.9, 111.4. HRMS-ESI (m/z): $[M+Na]^+$calcd for C13H9ClN2Na 251.0352; found 251.0351 [6].

2-(4-Nitrophenyl)-1*H*-benzimidazole (8a): Light yellow crystals; m.p. 312-314 °C. ^{1}H NMR (400 MHz, DMSO-d_6) δ ppm (J, Hz): 13.30 (br s, 1H), 8.40-8.37 (m, 4H), 7.67 (s, 2H), 7.27 (s, 2H); ^{13}C NMR (400 MHz, DMSO-d_6) δ ppm: 150.0, 149.0, 147.7, 136.0, 134.5, 127.32, 127.3, 124.14, 124.1, 122.9, 114.9. HRMS (ESI) Calc. for $C_{13}H_{10}N_3O_2$ $[M+H]^+$: 240.0770, found: 240.0768 [7].

4-(1*H*-Benzo[*d*]imidazol-2-yl)benzonitrile (9a): White solid; m.p. 235–236 °C. ^{1}H-NMR (400 MHz, DMSO-d_6) δ ppm (J, Hz): 8.18-8.16 (d, J = 8.1 Hz, 1H), 8.02-7.74 (m, 3H overlapping), 7.85-7.83 (d, J = 8.1 Hz, 2H overlapping), 7.44-7.42 (d, J = 8.1 Hz, 2H), 5.72 (s, 1H). ^{13}C-NMR (400 MHz, DMSO-d_6) δ ppm: 150.5, 139.7, 134.0, 133.1, 132.0, 131.0, 129.0, 128.0, 125.6, 119.0, 118.1, 116.2, 114.5, 111.6. HRMS (ESI) Calc. for $C_{14}H_9N_3Na$ $[M+Na]^+$: 242,0694, found: 242.0693 [5].

2-Phenyl-1*H*-benzimidazole (1a)

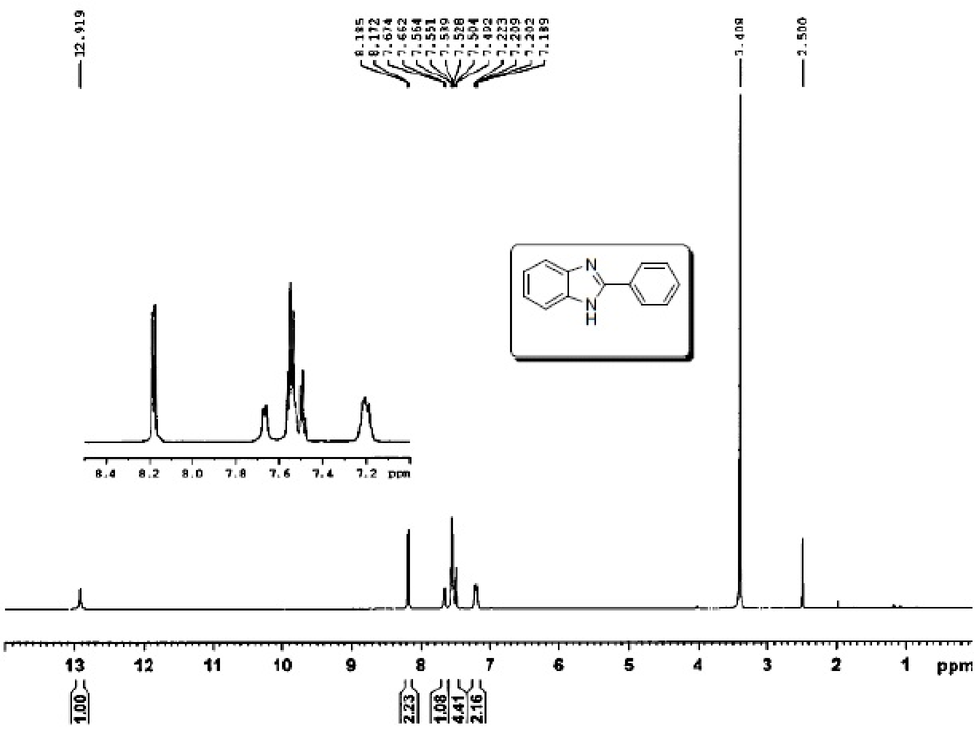

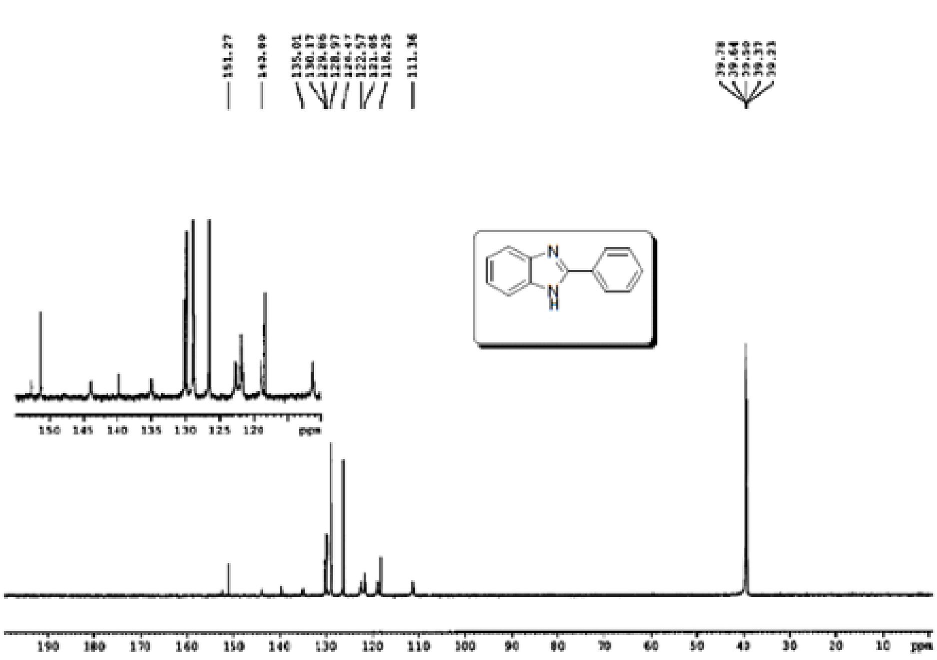

1-Benzyl-2-phenyl-1*H*-benzimidazole (1b)

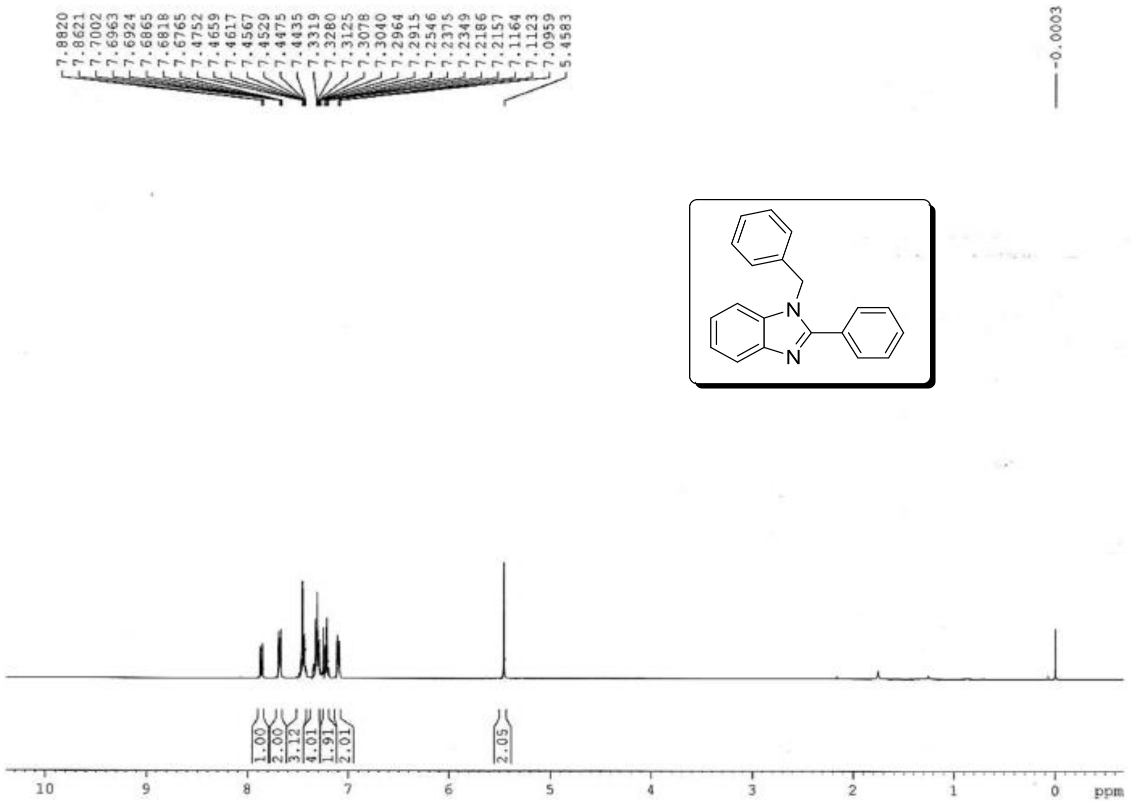

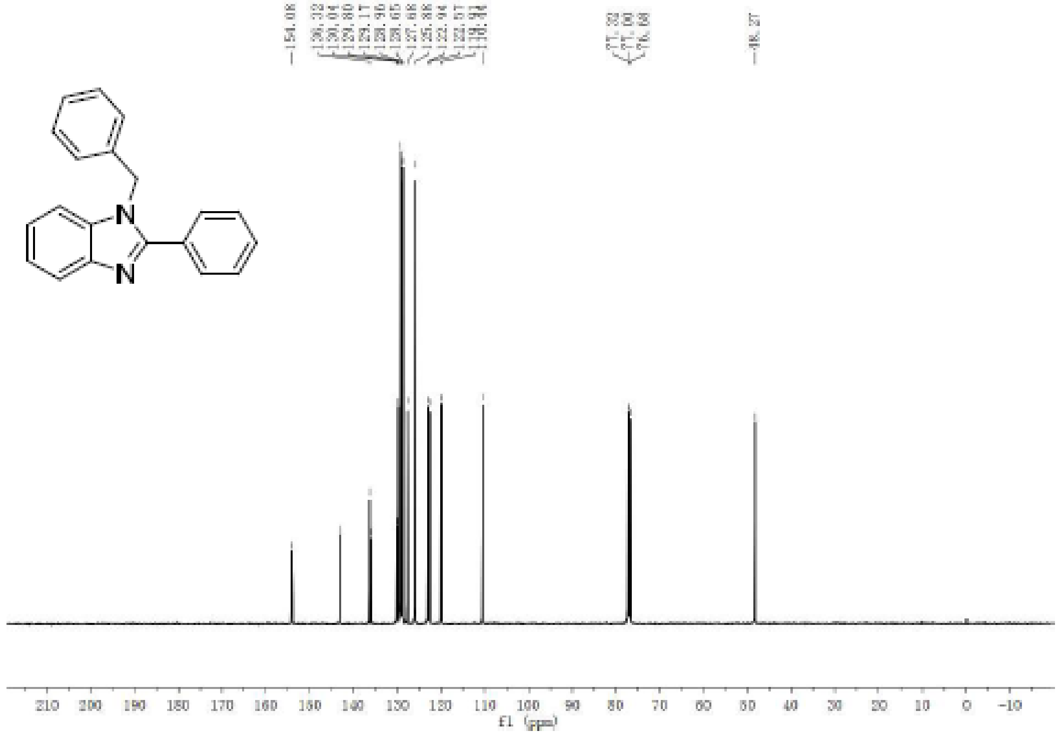

2-(4-Methoxyphenyl)-1*H*-benzimidazole (2a)

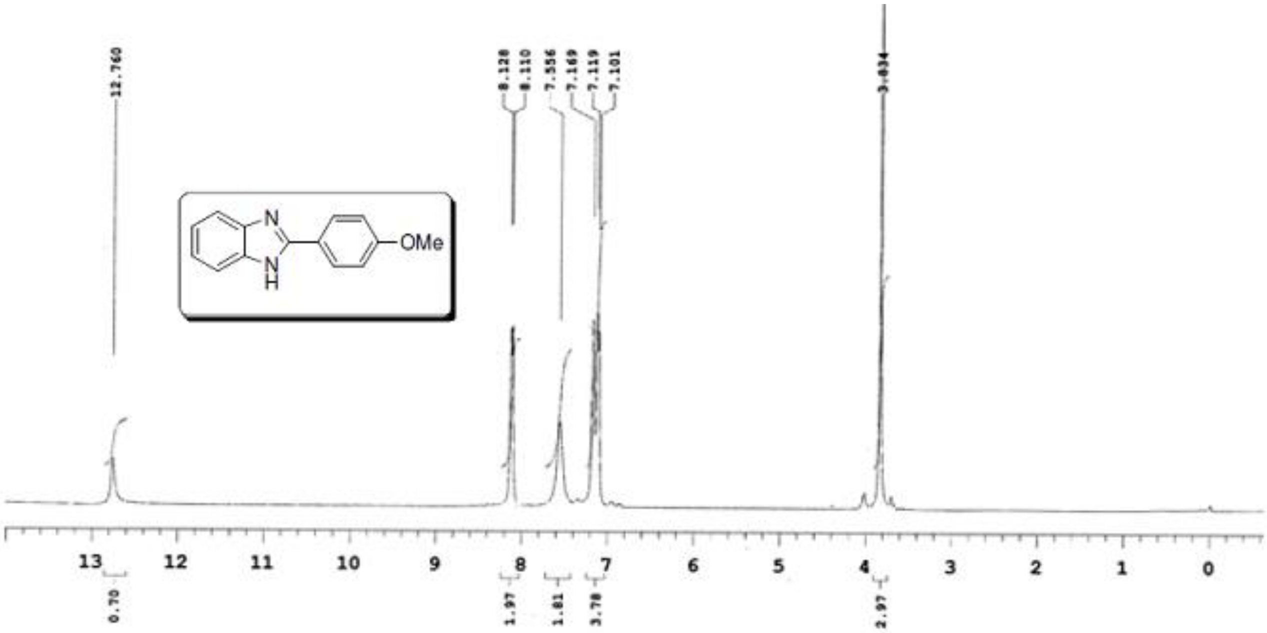

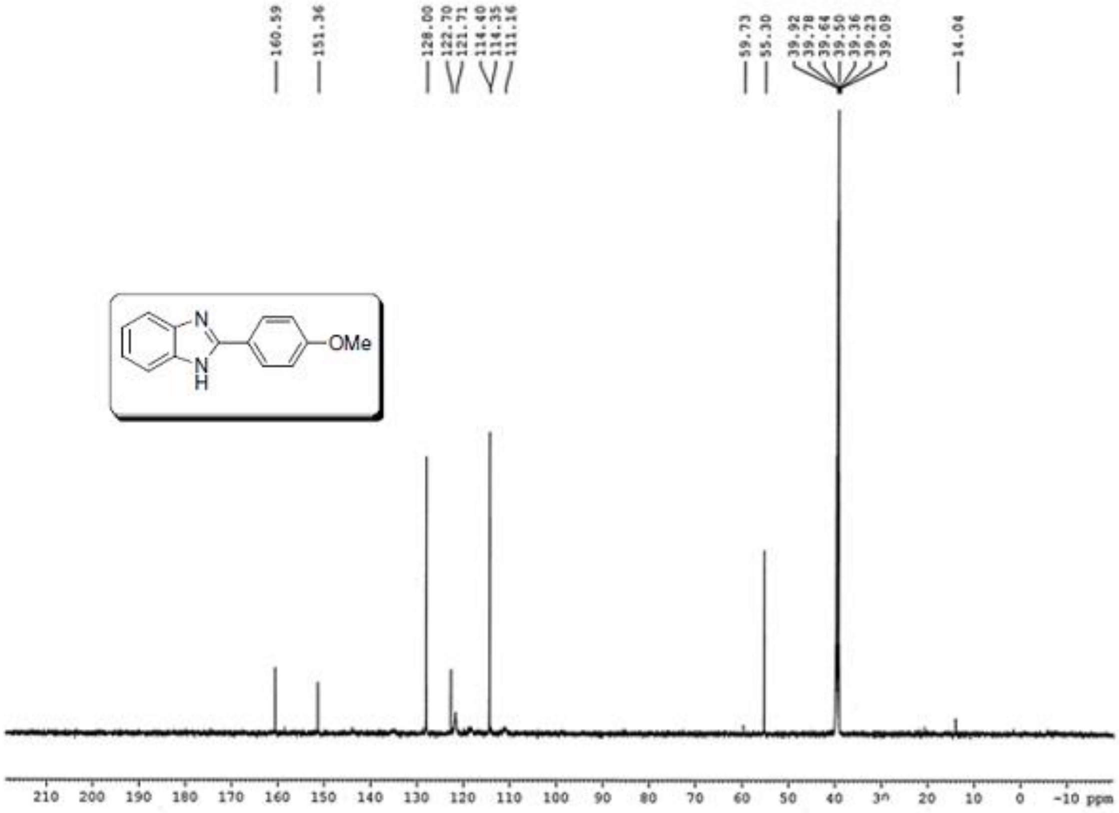

1-(4-Methoxybenzyl)-2-(4-methoxyphenyl)-1*H*-benzimidazole (2b)

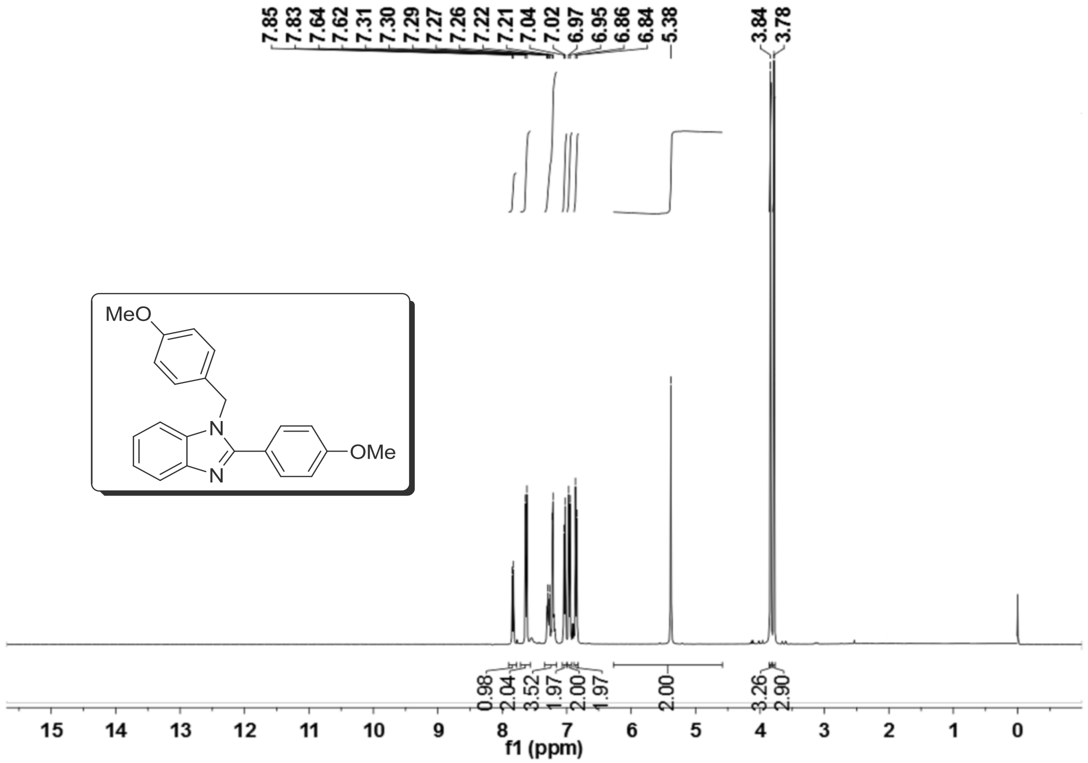

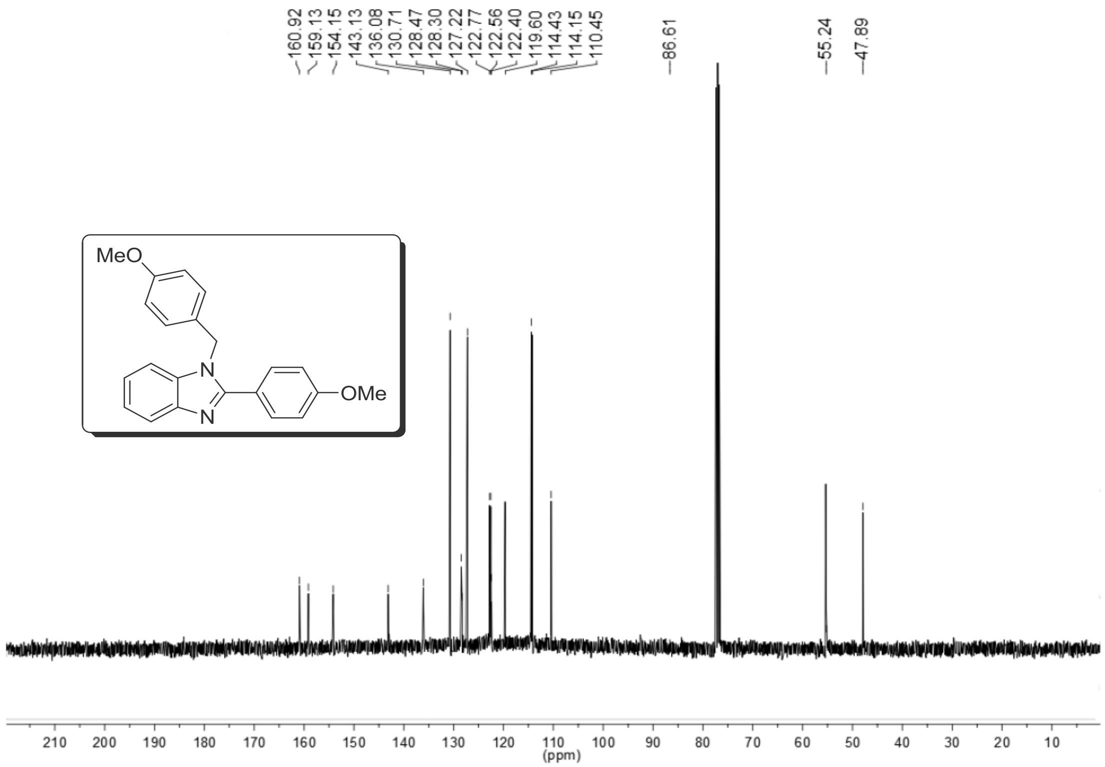

2-(4-Methylphenyl)benzimidazole (3a)

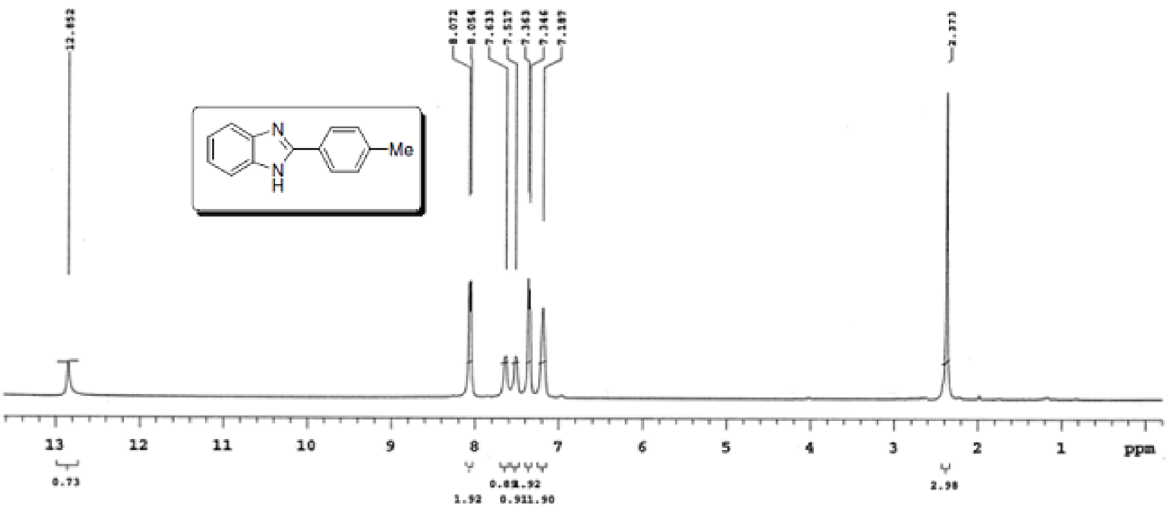

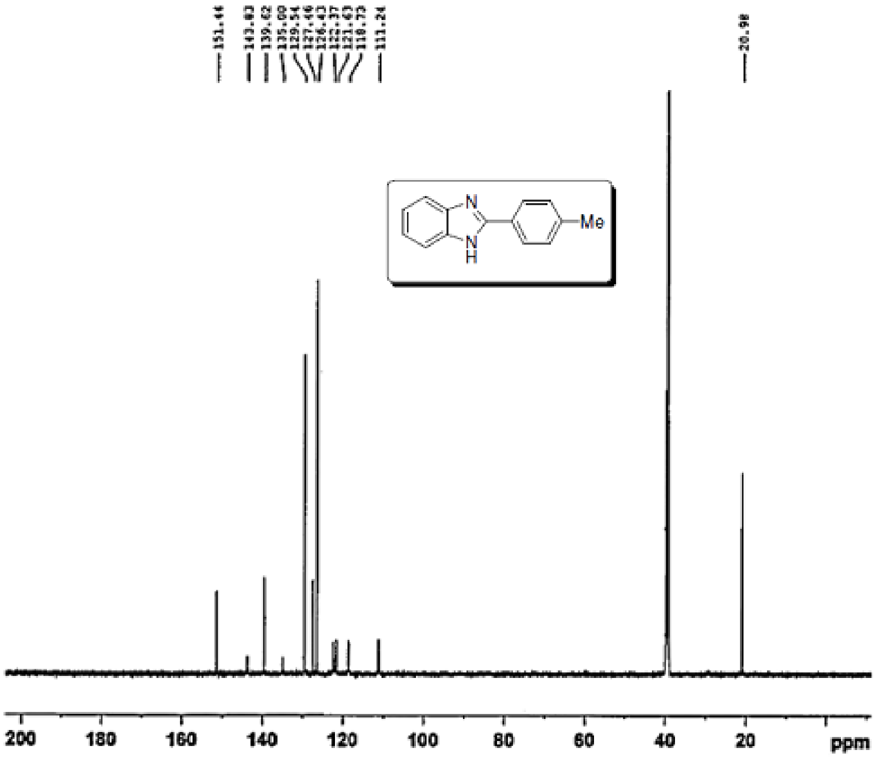

1-(4-Methylbenzyl)-2-(4-methylphenyl)-1*H*-benzimidazole (3b)

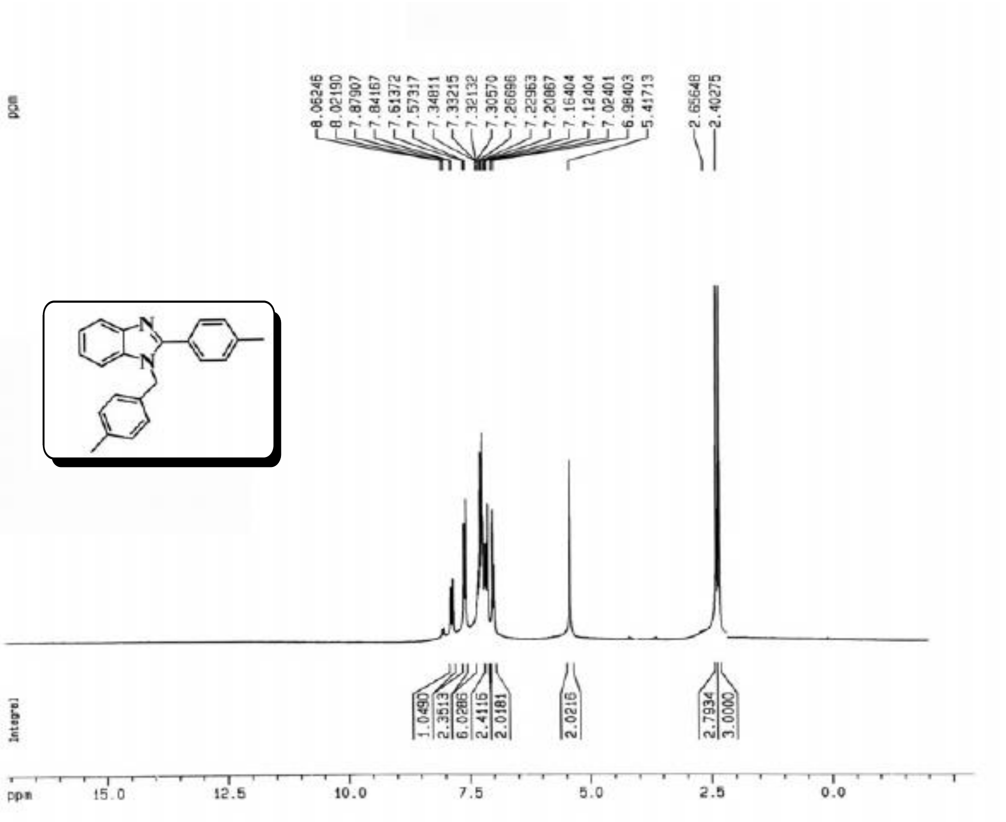

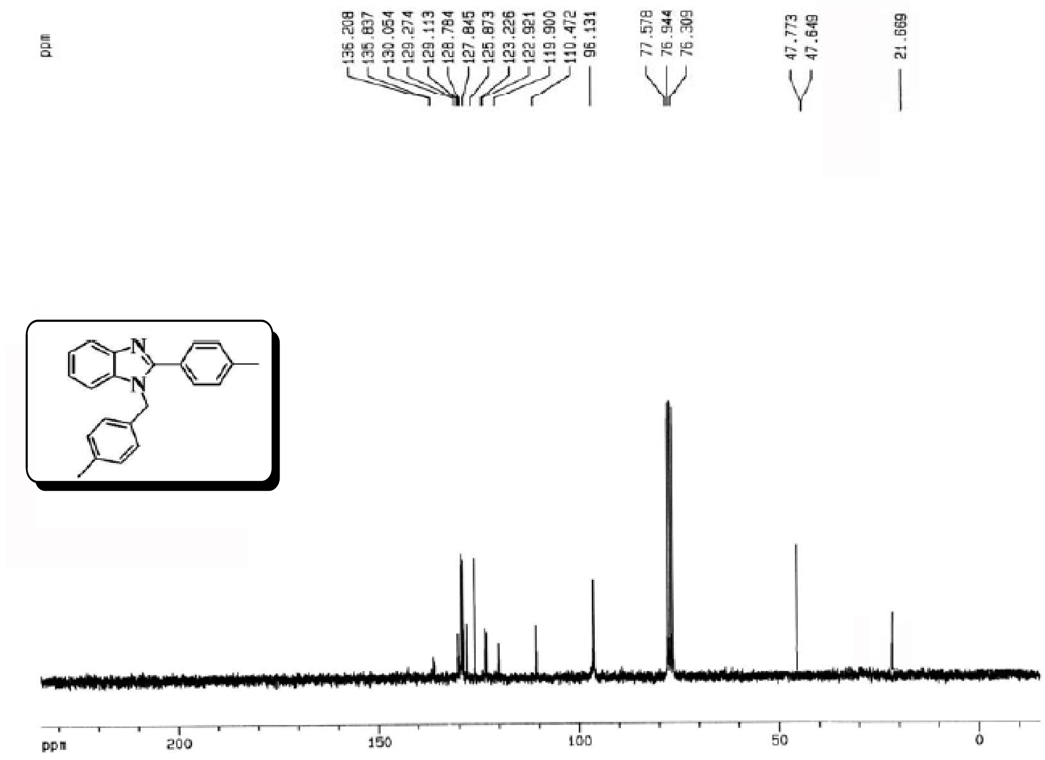

2-Ethylbenzimidazole (4a)

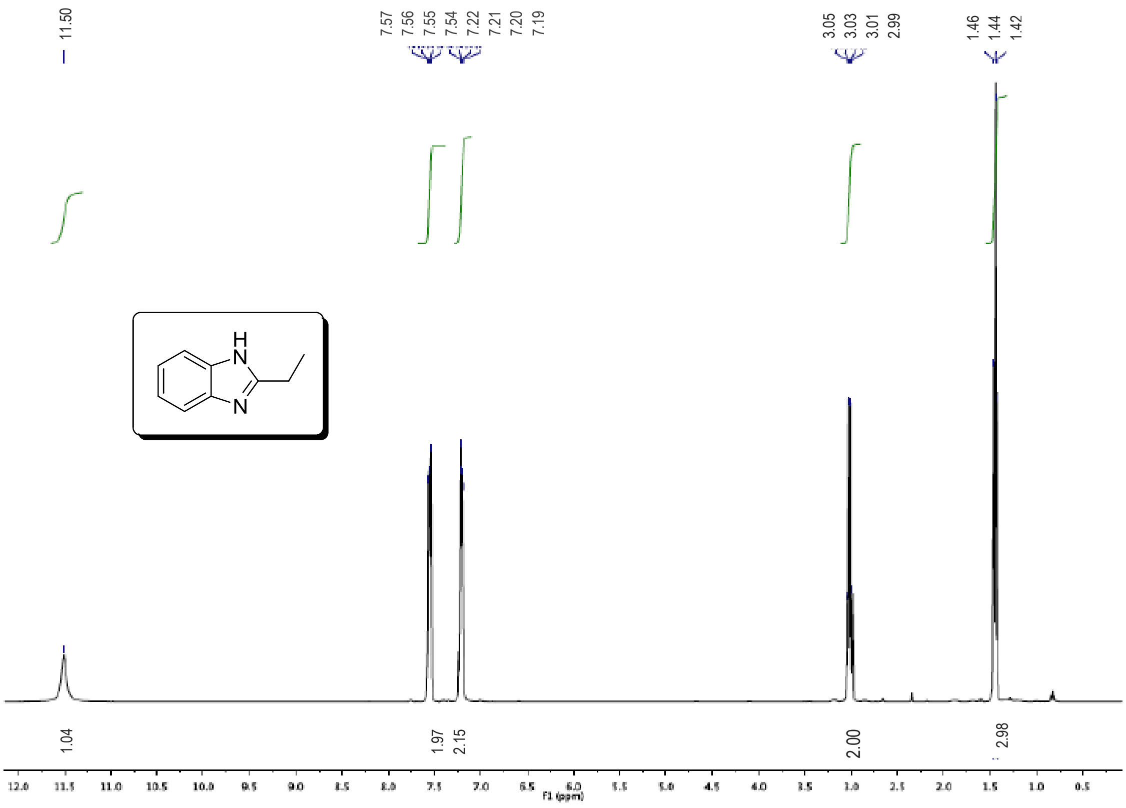

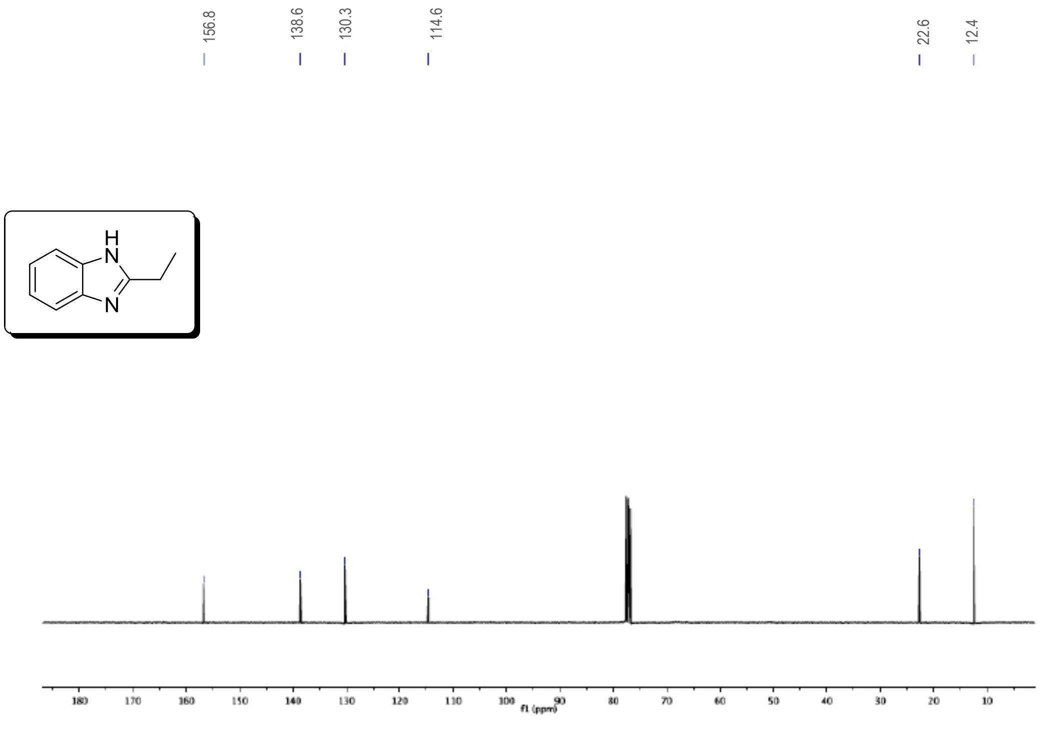

1-Ethyl-2-methyl-1H-benzo[*d*]imidazole (4b)

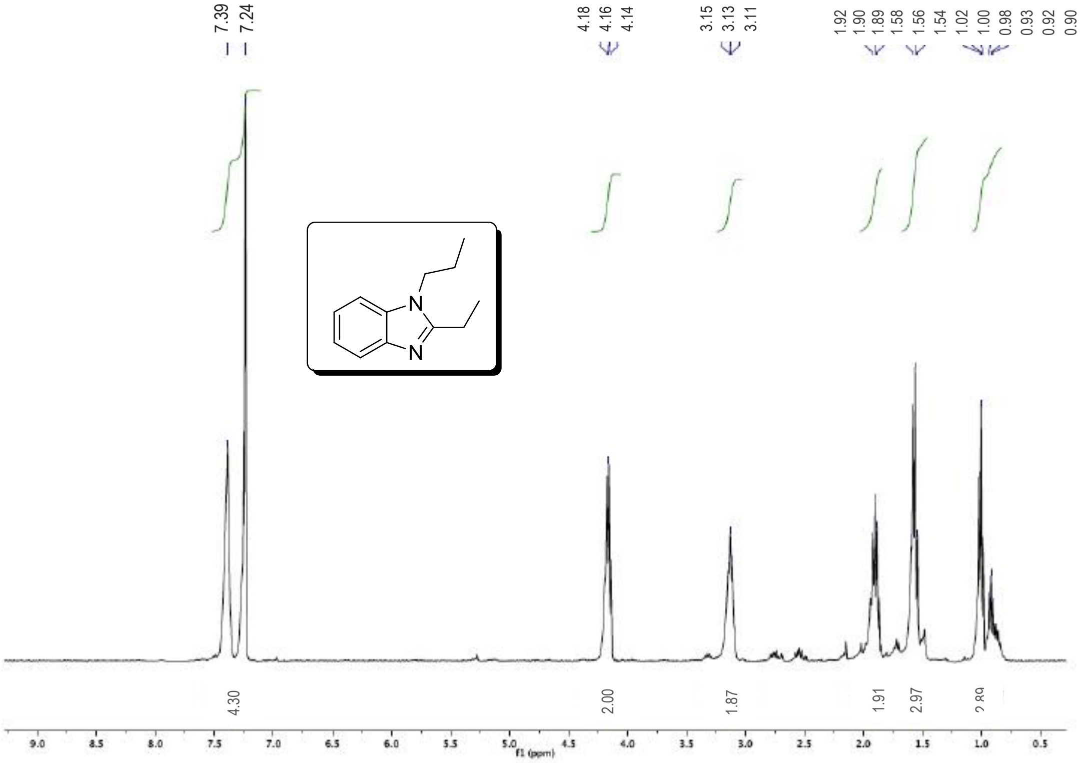

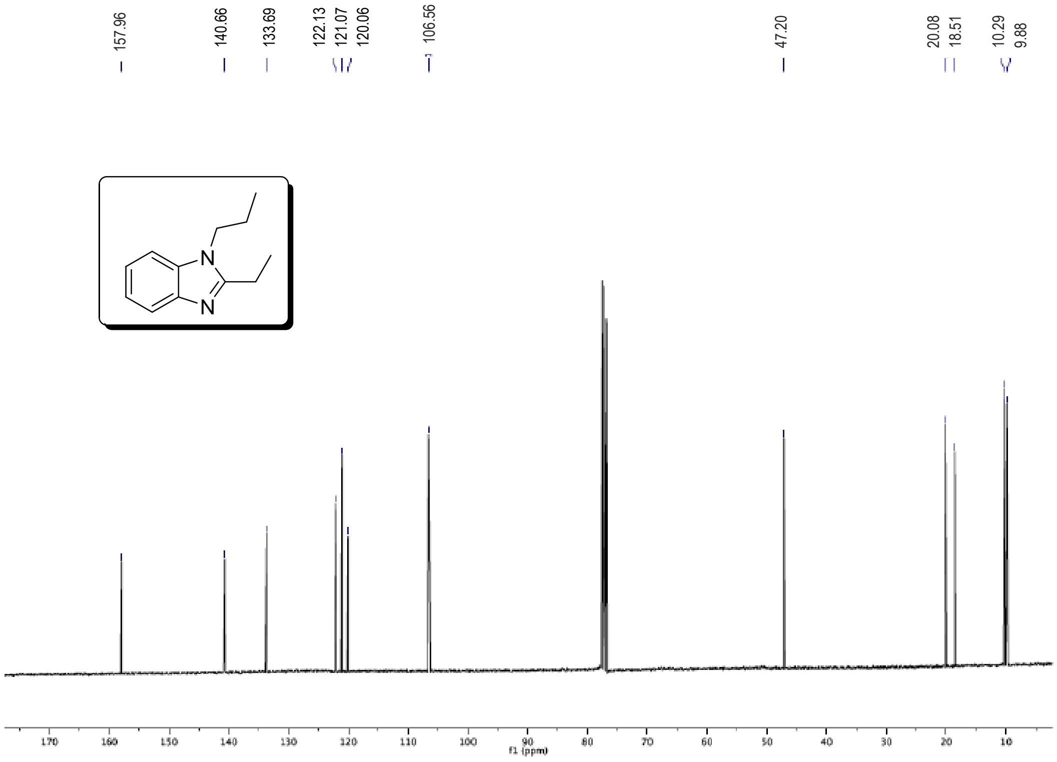

2-Methyl-1*H*-benzimidazole (5a)

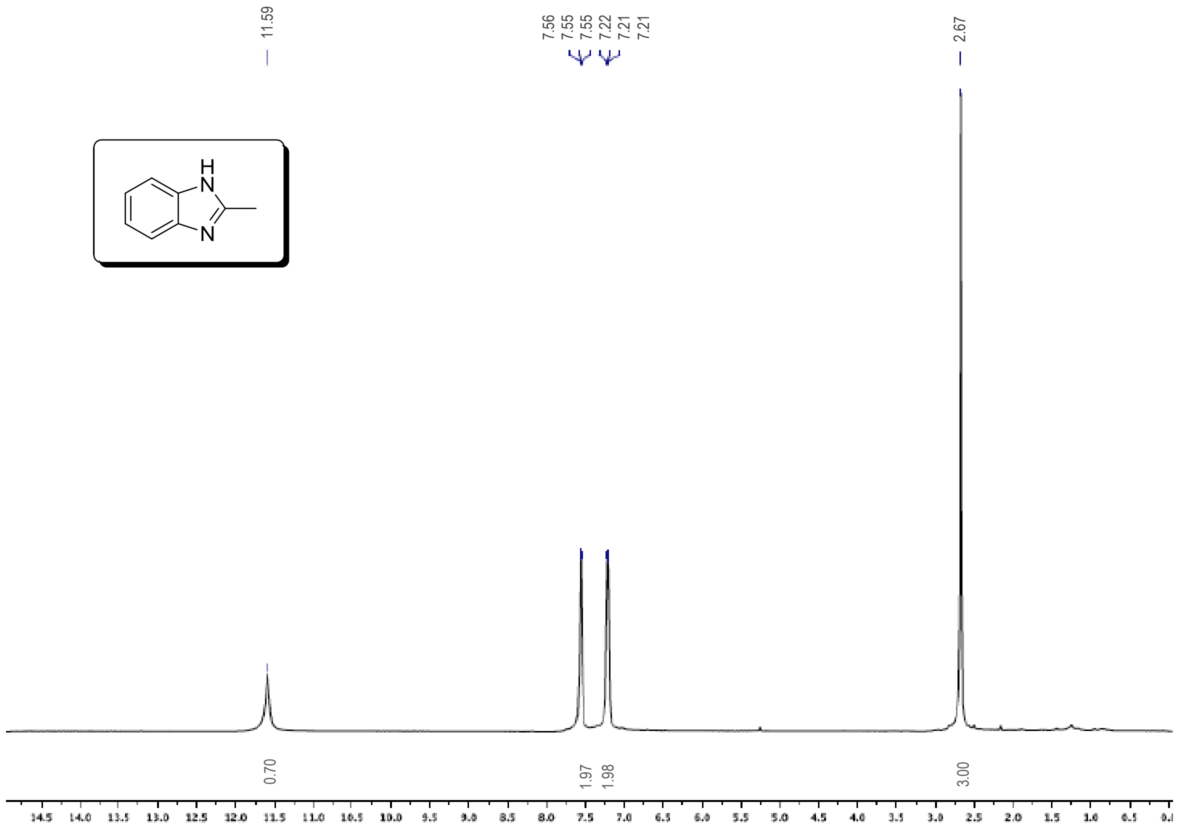

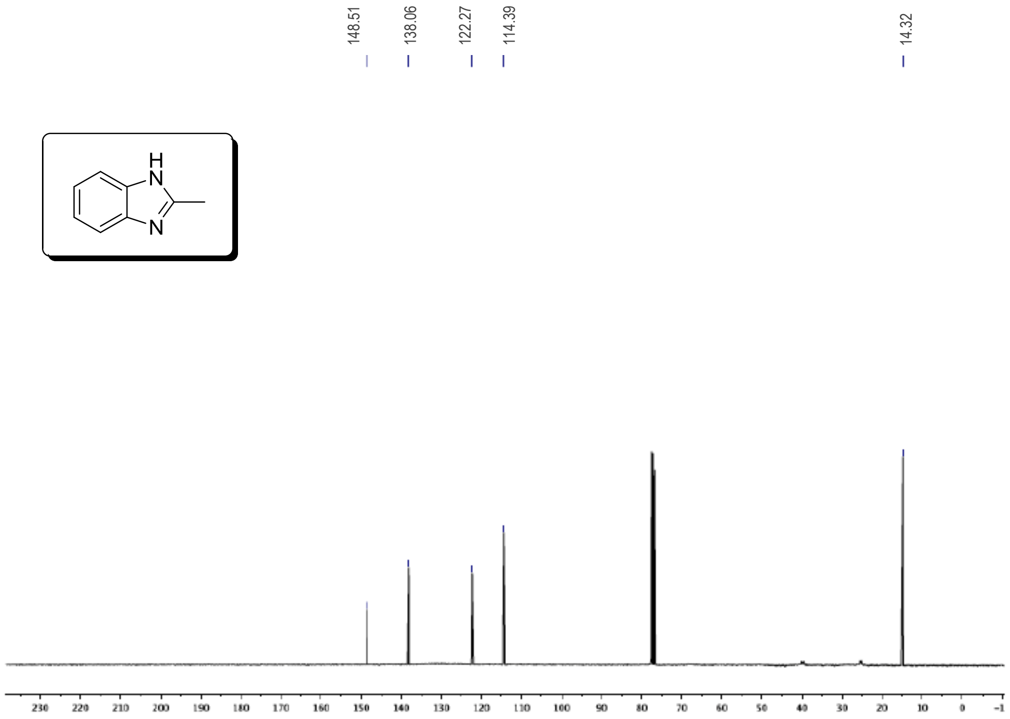

1-Ethyl-2-methyl-1*H*-benzo[d]imidazole (5b)

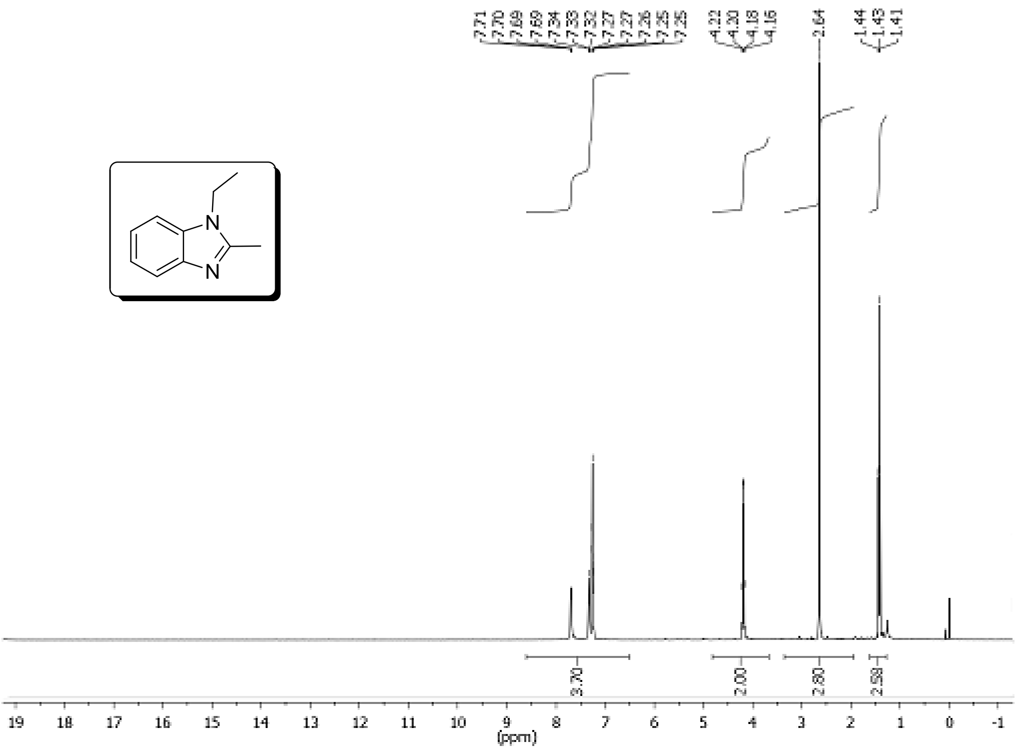

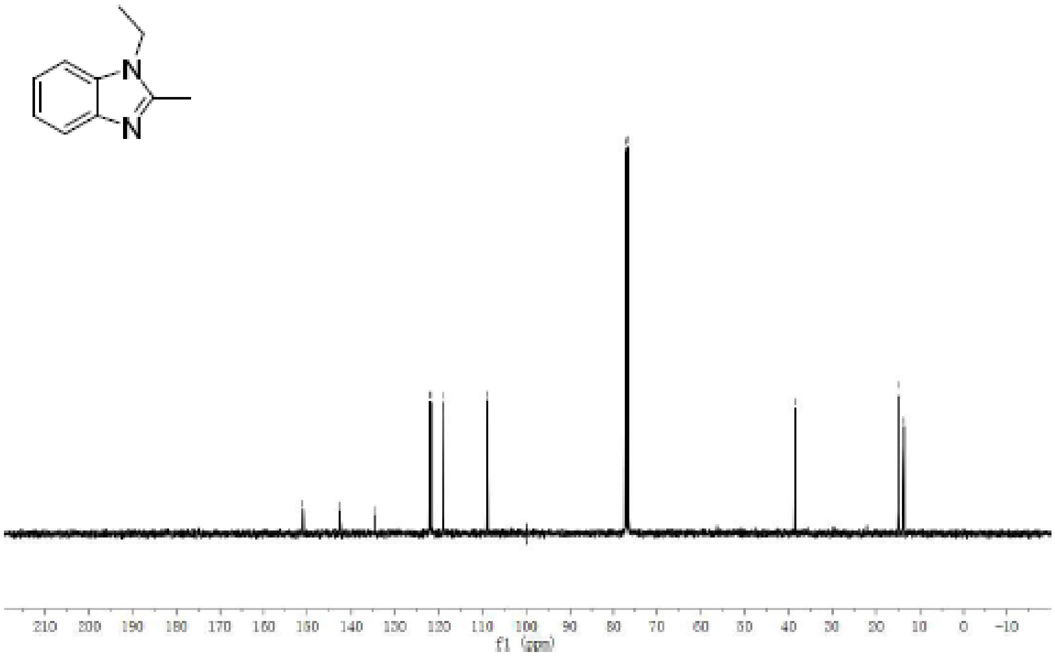

2-Benzyl-1*H*-benzimidazole (6a)

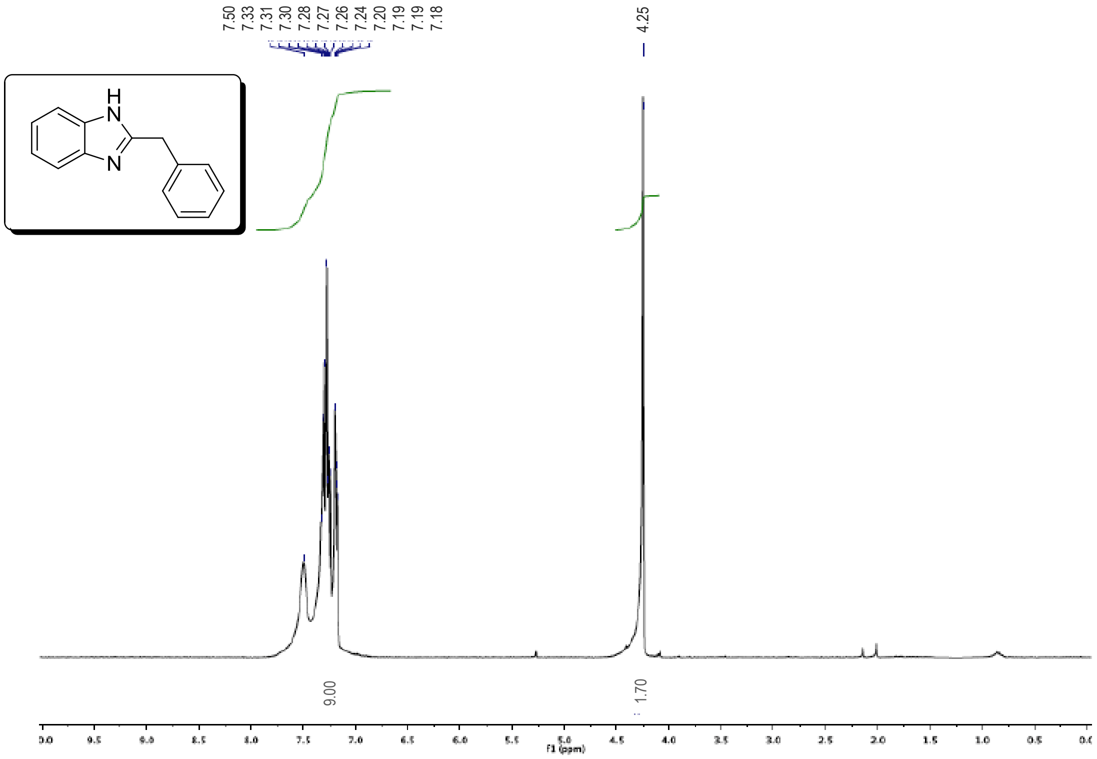

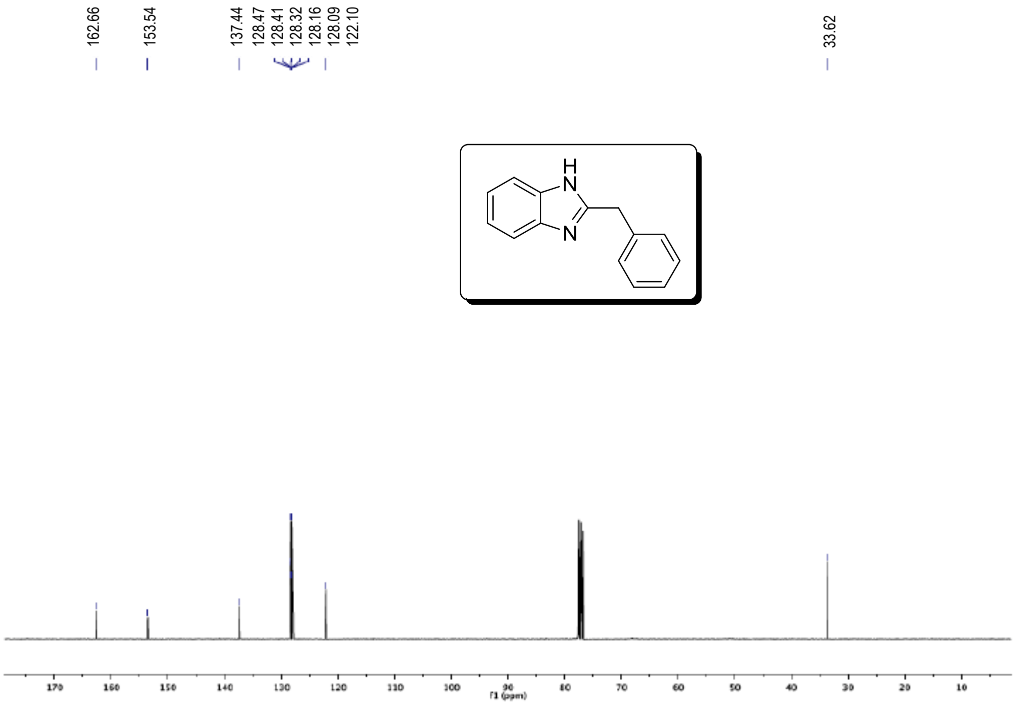

2-Benzyl-1-phenethyl-1*H*-benzimidazole (6b)

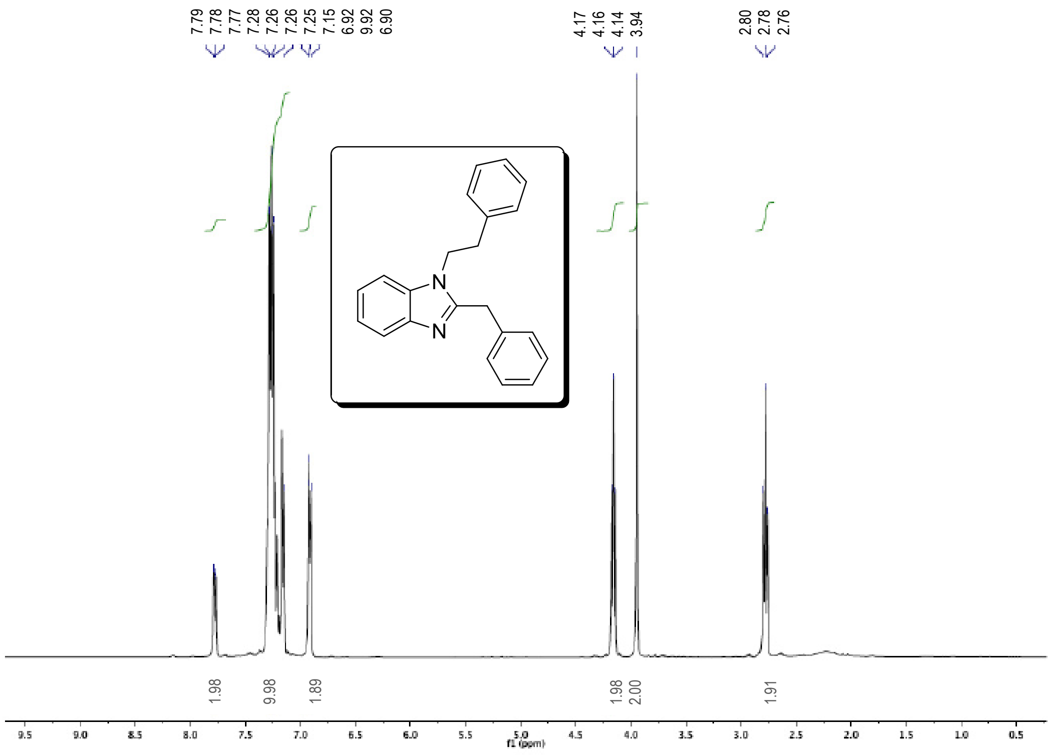

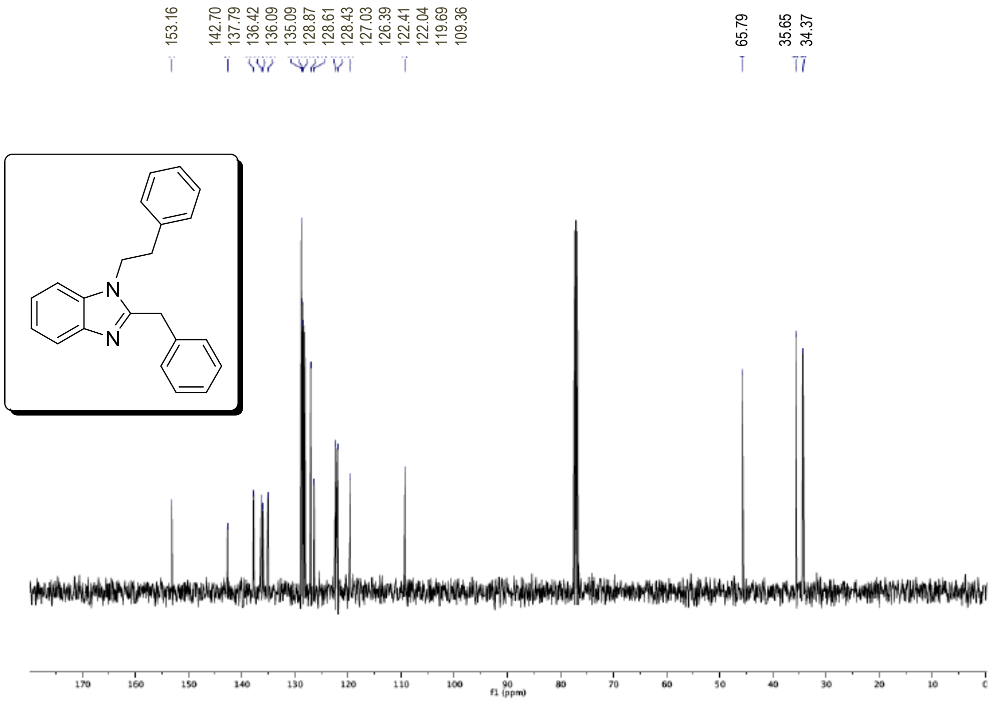

2-(4-Chlorophenyl)benzimidazole (7a)

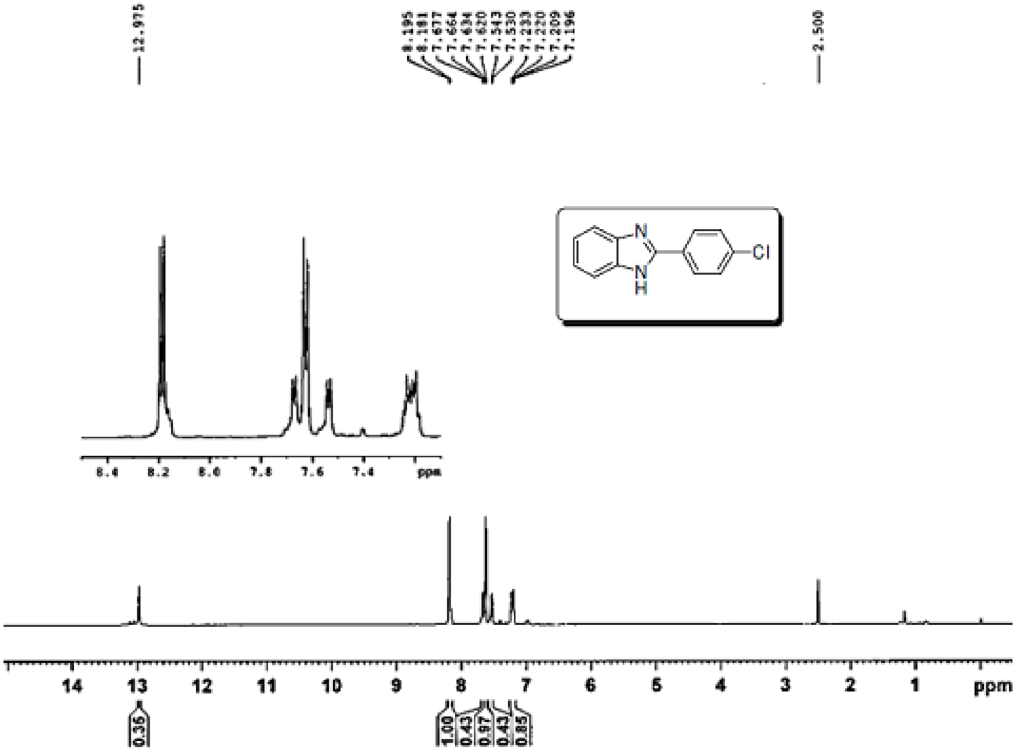

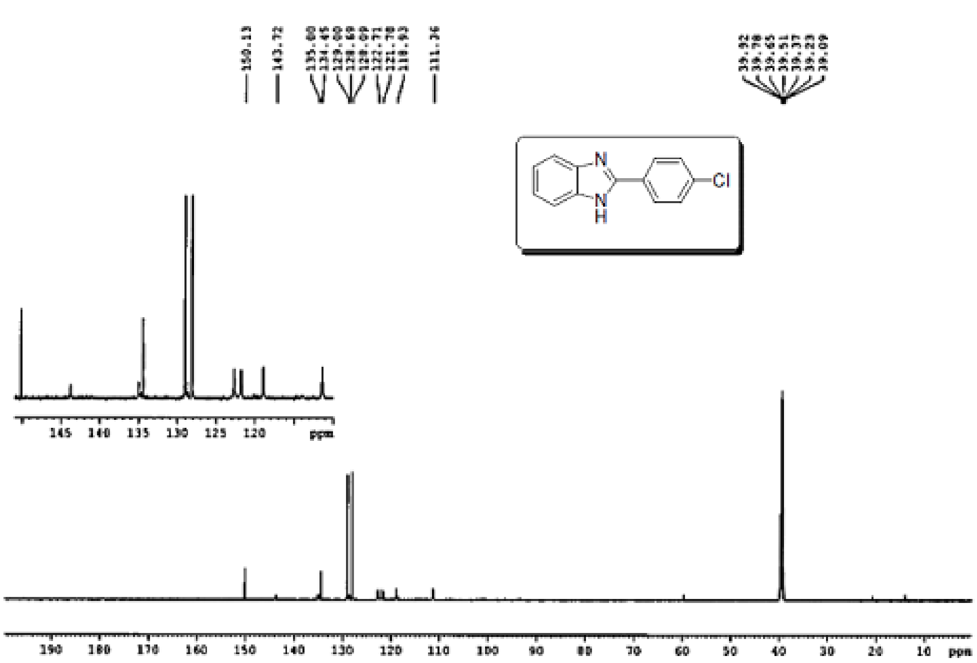

2-(4-Nitrophenyl)benzimidazole (8a)

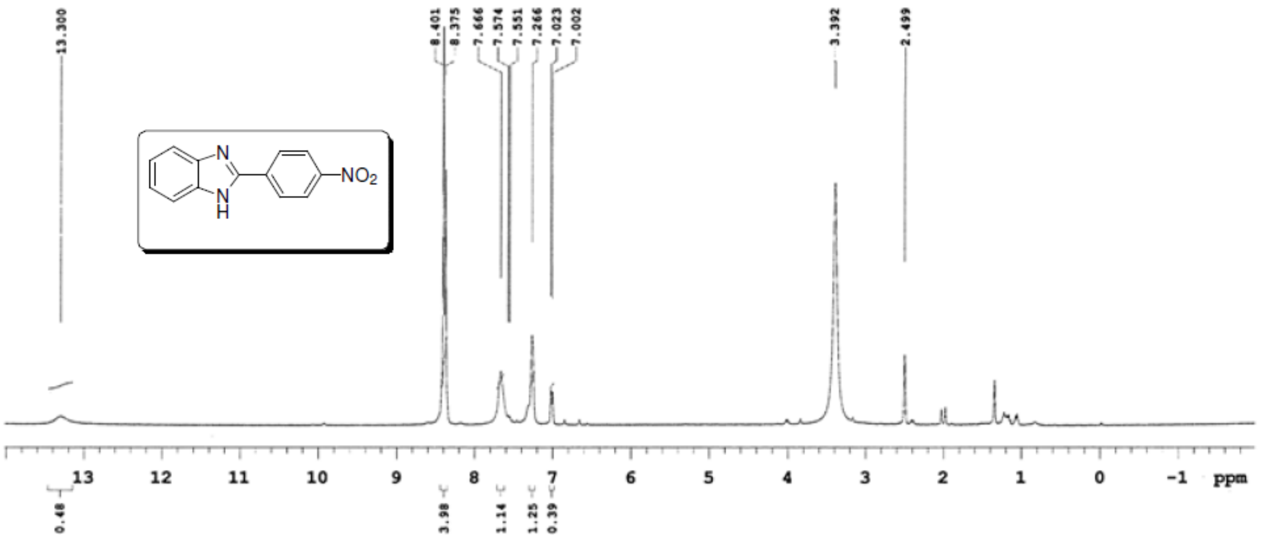

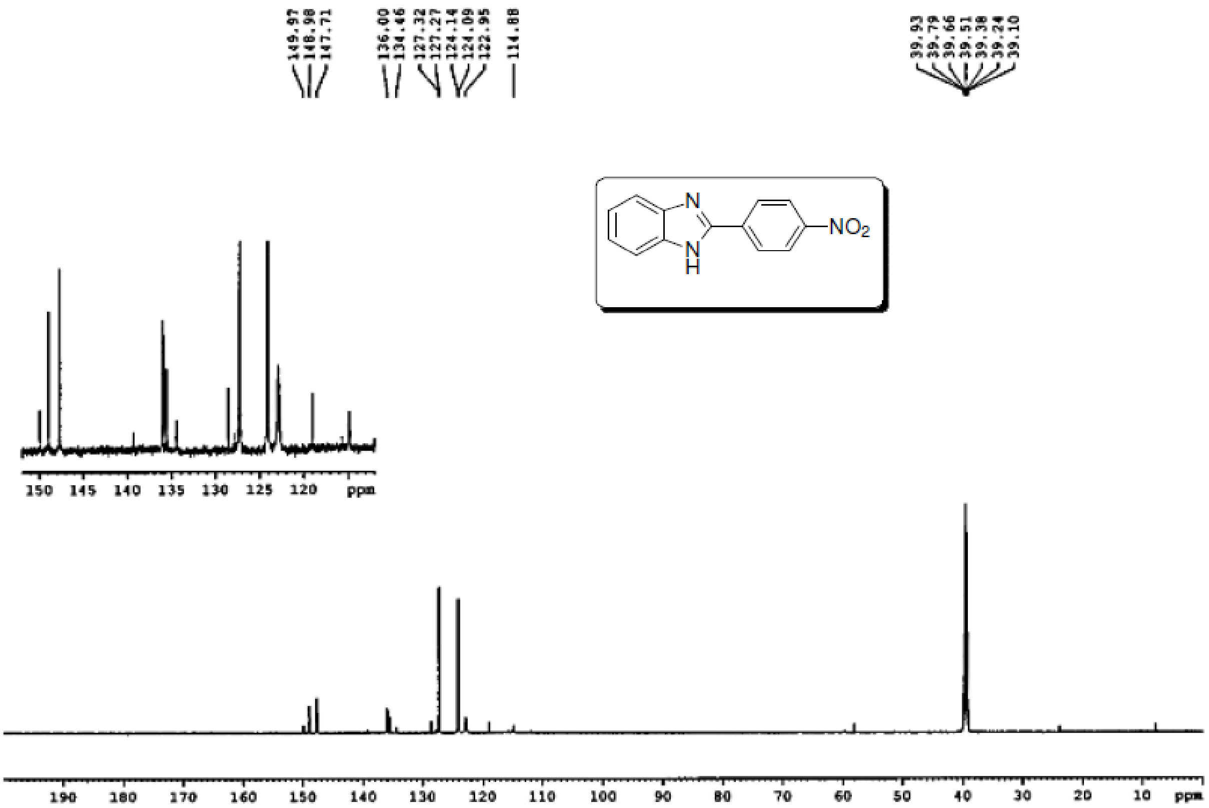

4-(1H-1,3-Benzimidazol-2-yl)benzonitrile (9a)

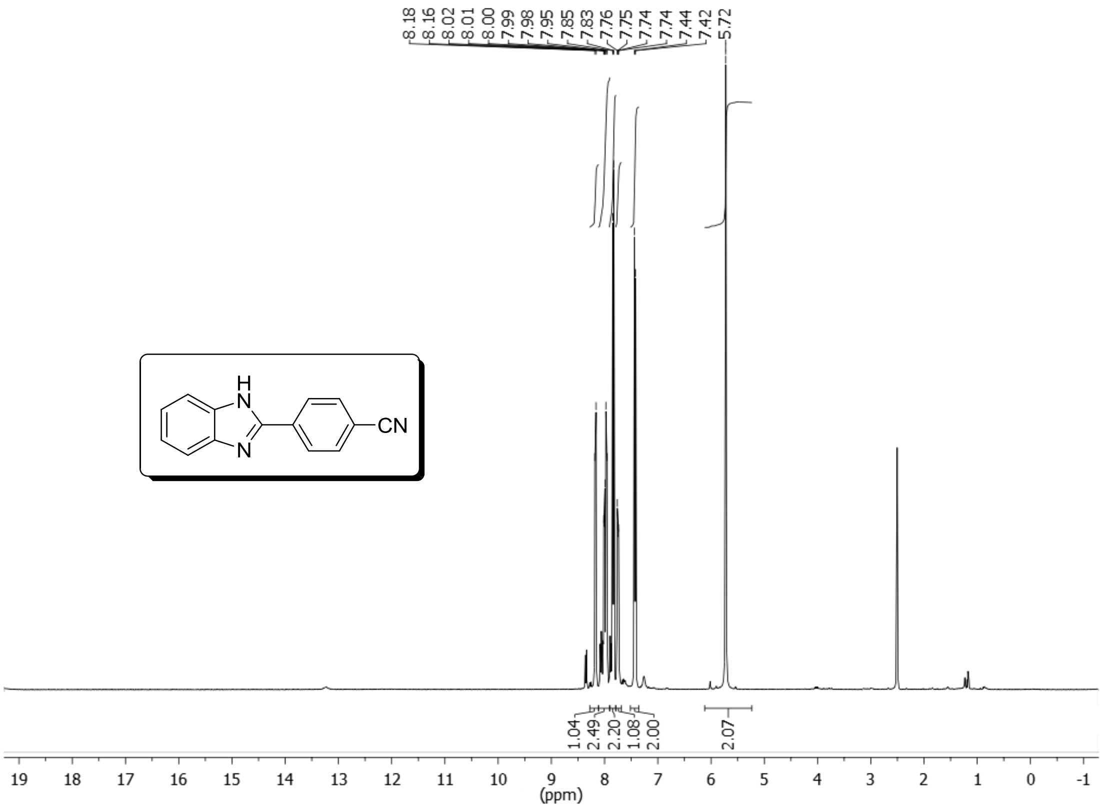

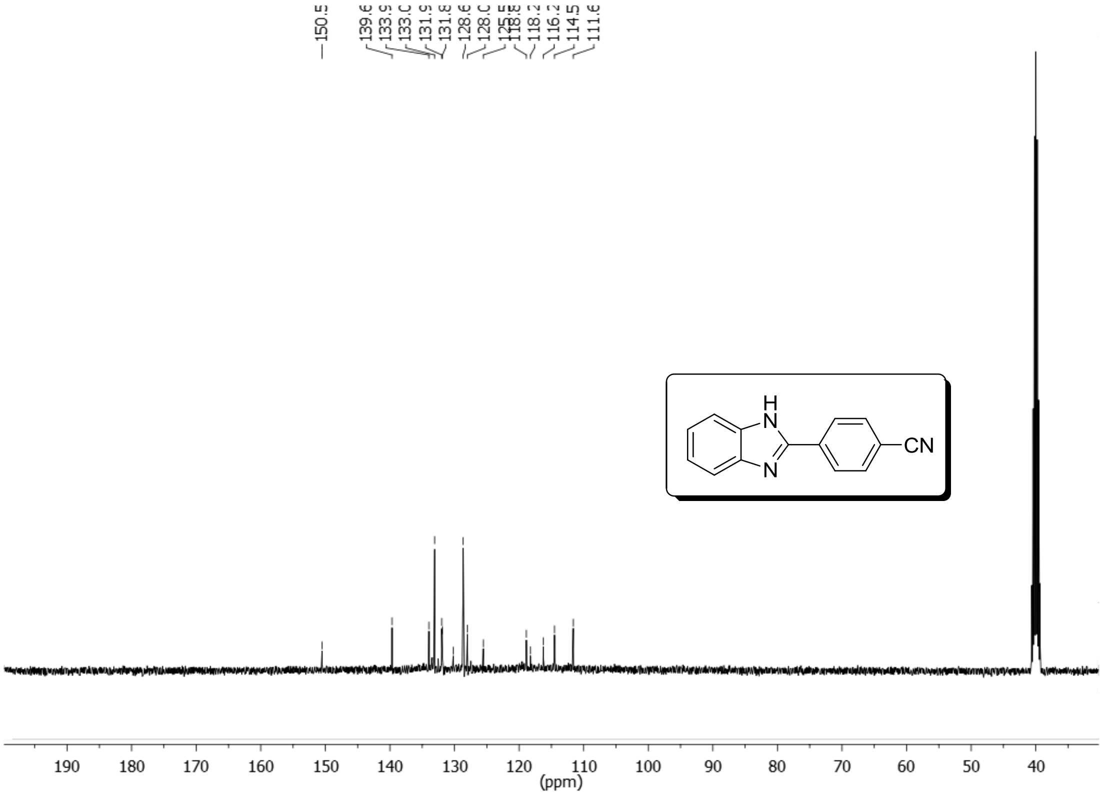

II. Cartesian Coordinates for aldehydes

Acetaldehyde

scf done: -153.825032

C	0.006161	0.000000	0.053018
O	0.092950	0.000000	1.279857
H	0.937143	0.000000	-0.565895
C	-1.282822	0.000000	-0.718645
H	-2.156860	0.000000	-0.052377
H	-1.309759	0.883002	-1.382479
H	-1.309759	-0.883002	-1.382479

Propanaldehyde

scf done: -193.130420

C	0.022324	-0.047811	0.083336
O	0.126401	-0.159774	1.303752
H	0.946017	0.024637	-0.545299
C	-1.277810	0.005009	-0.678763
H	-2.120218	-0.020567	0.030707
C	-1.350279	1.230713	-1.614694
H	-1.322184	-0.916629	-1.292584
H	-2.272129	1.192877	-2.215520
H	-1.354816	2.170575	-1.039674
H	-0.494708	1.256650	-2.309768

2-Phenylacetaldehyde

scf done: -384.851769

C	-0.123825	-0.619262	-0.529241
O	0.745515	-0.911744	0.286390
H	-0.099344	-1.056432	-1.557560
C	-1.278274	0.332726	-0.259350
H	-1.168715	0.748757	0.754072
C	-1.345984	1.422110	-1.320071
H	-2.203867	-0.273265	-0.286537
C	-2.101882	1.245750	-2.497215
C	-2.131811	2.243637	-3.485537

C	-1.405153	3.433088	-3.309621
C	-0.646578	3.617188	-2.140885
C	-0.614186	2.617037	-1.155875
H	-2.679271	0.326216	-2.637853
H	-2.728540	2.092877	-4.390065
H	-1.431798	4.212624	-4.076558
H	-0.079628	4.541290	-1.993966
H	-0.021839	2.767665	-0.247592

Benzaldehyde

scf done: -345.551480

C	0.046875	-0.000000	-0.066120
C	0.004607	-0.000000	1.347340
C	1.195788	0.000000	2.080316
C	2.435792	0.000000	1.410713
C	2.485297	0.000000	0.005754
C	1.293111	0.000000	-0.731303
H	-0.965394	-0.000000	1.851701
H	1.165552	0.000000	3.173446
H	3.364884	0.000000	1.987904
H	3.449496	0.000000	-0.509708
H	1.320714	0.000000	-1.826131
C	-1.191256	-0.000000	-0.872933
O	-2.340353	-0.000000	-0.418471
H	-1.030366	-0.000000	-1.978765

4-Chlorobenzaldehyde

scf done: -805.150396

C	0.047030	-0.000000	-0.068031
C	0.009440	-0.000000	1.344885
C	1.194164	-0.000000	2.084313
C	2.424091	0.000000	1.398924
C	2.489626	0.000000	-0.004082
C	1.293533	0.000000	-0.730648
H	-0.956364	-0.000000	1.856613
H	1.172807	-0.000000	3.175954
Cl	3.916398	0.000000	2.325213

H	3.455236	0.000000	-0.513439
H	1.327504	0.000000	-1.824651
C	-1.192585	-0.000000	-0.871866
O	-2.338740	-0.000000	-0.411149
H	-1.034950	-0.000000	-1.977796

4-Formylbenzonitrile

scf done: -437.794180

C	0.060564	-0.000000	-0.121292
C	0.022027	-0.000000	1.289092
C	1.225331	0.000000	2.029096
C	2.454112	0.000000	1.370015
C	2.485218	0.000000	-0.047645
C	1.284808	-0.000000	-0.795699
C	-1.296158	-0.000000	1.972622
O	-1.451194	0.000000	3.195365
C	3.747148	0.000000	-0.730225
N	4.782386	0.000000	-1.288875
H	1.182598	0.000000	3.120796
H	3.388693	0.000000	1.934585
H	1.320752	-0.000000	-1.886746
H	-0.873029	-0.000000	-0.691746
H	-2.180914	-0.000000	1.292434

4-Methylbenzaldehyde

scf done: -384.863333

C	0.018119	0.000000	-0.086738
C	-0.003382	0.000000	1.328457
C	1.192551	0.000000	2.046608
C	2.445505	-0.000000	1.383816
C	2.457790	-0.000000	-0.027196
C	1.261633	-0.000000	-0.755262
H	-0.965346	0.000000	1.848344
H	1.166322	0.000000	3.141003
C	3.729827	-0.000000	2.179380
H	3.414780	-0.000000	-0.557698
H	1.286549	-0.000000	-1.850258

C	-1.222433	0.000000	-0.881748
O	-2.371143	0.000000	-0.421497
H	-1.068499	-0.000000	-1.989002
H	4.614345	-0.000000	1.524756
H	3.787845	0.885722	2.835389
H	3.787845	-0.885722	2.835389

4-Methoxybenzaldehyde

scf done: -460.071567

C	-0.032910	-0.000000	-0.094403
C	0.010453	-0.000000	1.324244
C	1.227581	-0.000000	1.993222
C	2.442540	0.000000	1.258797
C	2.417249	0.000000	-0.154027
C	1.183069	0.000000	-0.812453
H	-0.926910	-0.000000	1.886983
H	1.274722	-0.000000	3.085175
O	3.576810	0.000000	2.010118
H	3.340229	0.000000	-0.735364
H	1.161270	0.000000	-1.907408
C	-1.301086	-0.000000	-0.832062
O	-2.432811	-0.000000	-0.325001
H	-1.194159	0.000000	-1.945020
C	4.852163	0.000000	1.336991
H	5.601359	0.000000	2.137951
H	4.968955	-0.903988	0.717393
H	4.968955	0.903988	0.717393

4-Nitrobenzaldehyde

scf done: -550.069570

C	0.047800	-0.000000	-0.073059
C	0.007508	-0.000000	1.339043
C	1.193447	0.000000	2.075038
C	2.414076	0.000000	1.378132
C	2.485439	0.000000	-0.022606

C	1.288299	-0.000000	-0.745763
H	-0.958622	-0.000000	1.848658
H	1.184745	0.000000	3.165293
N	3.674818	0.000000	2.152432
H	3.453176	0.000000	-0.524551
H	1.316592	-0.000000	-1.839144
C	-1.205672	-0.000000	-0.873130
O	-2.339408	-0.000000	-0.392023
H	-1.061855	-0.000000	-1.979432
O	4.748535	0.000000	1.521396
O	3.597357	0.000000	3.395416

IV. References

[1]. Du, L-H.; Wang, Y-G. *Synthesis* **2007**, *5,* 675- 678.

[2]. Jacob, R. G.; Dutra, L. G.; Radatz, C. S.; Mendes, S. R.; Perin, G.; Lenardao, E. J. *Tetrahedron Lett*. **2009**, *50*, 1495–1497.

[3]. Chen, C.; Chen, C.; Li, B.; Tao, J.; Peng, J. *Molecules* **2012**, *17,* 12506-12520.

[4]. Pizzetti, M.; De Luca, E.; Petricci, E.; Porcheddu, A.; Taddei, M. *Adv. Synth. Catal*. **2012**, *354*, 2453–2464.

[5]. Sun, X.; Lv, X-H.; Ye,L-M.; Hu, Y.; Chen, Y-Y.; Zhang, X-J.; Yan, M. *Org. Biomol. Chem*. **2015**, *13*, 7381-7383.

[6]. Peng, J.; Ye, M.; Zong, C.; Hu, F.; Feng, L.; Wang, X.; Wang, Y.; Chen. Ch. *J. Org. Chem*., 2011, *76*, 716–719.

[7]. Chen, G-F.; Shen, H-D.; Jia, H-M.; Zhang, L-Y.; Kang, H-Y.; Qi, Q-Q.; Chen, B-H.; Cao, J-L.; Li, J-T. *Aust. J. Chem*. **2013**, *66*, 262–266.
